# Comparison of phenotypic and transcriptomic profiles between HFPO-DA and prototypical PPARα, PPARγ, and cytotoxic agents in wild-type and *Ppara*-null mouse livers

**DOI:** 10.1093/toxsci/kfaf049

**Published:** 2025-04-11

**Authors:** Melissa M Heintz, Amanda N Buerger, Laurie C Haws, John M Cullen, Alexander W East, Chad M Thompson

**Affiliations:** ToxStrategies LLC, Asheville, NC 28801, United States; ToxStrategies LLC, Asheville, NC 28801, United States; ToxStrategies LLC, Austin, TX 78731, United States; Hagler Fellow, Texas A&M University, Raleigh, NC 27607, United States; ToxStrategies LLC, Asheville, NC 28801, United States; ToxStrategies LLC, Katy, TX 77494, United States

**Keywords:** HFPO-DA (GenX), PFAS, peroxisome proliferator-activated receptor alpha (PPARα), mode of action (MOA), liver, transcriptomics

## Abstract

Recent in vitro transcriptomic analyses for short-chain per- and polyfluoroalkyl substances HFPO-DA (ammonium, 2,3,3,3-tetrafluoro-2-(heptafluoropropoxy)-propanoate) added to the weight of evidence supporting the peroxisome proliferator-activated receptor alpha (PPARα) activator-induced hepatocarcinogenesis mode of action (MOA) for HFPO-DA-mediated liver effects in rodents. Importantly, PPARα-mediated key events (KEs) including hepatocellular hypertrophy and proliferation that have been shown to occur prior to tumor development in this MOA are rodent-specific and likely not human-relevant. To further inform the MOA of HFPO-DA and evaluate other hypothesized MOAs, phenotypic and transcriptomic responses in wild-type (WT) and *Ppara*-null mice were investigated following short-term exposure to HFPO-DA or prototypical agonists of PPARα (GW7647), PPARγ (rosiglitazone), or cytotoxicity (acetaminophen). Phenotypic and transcriptomic assessment of mouse livers demonstrated a general lack of response to HFPO-DA or GW7647 exposure in *Ppara*-null but not WT mice. Conversely, rosiglitazone or acetaminophen elicited similar phenotypic and transcriptomic responses between genotypes demonstrating a lack of PPARα-dependence. In WT mice, HFPO-DA-mediated responses were similar to GW7647 but different from rosiglitazone or acetaminophen. Dose-dependent increases in liver weight, karyomegaly, and mitosis, as well as increased transcriptomic signaling related to PPARα activation and cell proliferation were observed in HFPO-DA and GW7647-exposed WT mice. The consistent phenotypic and transcriptomic signaling patterns between HFPO-DA and GW7647 in WT mice, and the lack of changes in *Ppara*-null mice, provide further support that HFPO-DA-mediated early KEs in mouse liver are PPARα-dependent, occur via the rodent-specific PPARα MOA, and therefore are not appropriate for use in human health risk assessment.

The short-chain polyfluorinated ether, HFPO-DA (ammonium, 2,3,3,3-tetrafluoro-2-(heptafluoropropoxy)-propanoate; CASRN 62037-80-3) is a major component of the GenX polymer processing aid used in the manufacture of certain types of fluorinated polymers and is part of the broader per- and polyfluoroalkyl substances (PFAS) chemical group. Like other PFAS, toxicity studies indicate that the liver is the primary target of toxicity in rodents following oral exposure to HFPO-DA (Thompson et al. 2019; USEPA 2021). However, due to the large diversity in PFAS chemical structures, including differences in carbon chain lengths, functional groups, and interchain linkages, the modes of action (MOAs) that mediate liver effects observed following PFAS exposure may differ between individual PFAS.

In human health risk assessment, consideration of a chemical’s MOA reduces uncertainties and informs human relevance. Based on the available MOA studies for HFPO-DA, the current weight of the evidence (WOE) supports that liver effects observed in rodents following exposure to HFPO-DA are occurring via the peroxisome proliferator-activated receptor alpha (PPARα) MOA (Chappell et al. 2020; Heintz et al. 2022, 2023). Briefly, this well-established MOA (which is also under development as an adverse outcome pathway) for liver tumors in rodents (the adverse outcome) includes 4 key events (KEs): (KE 1) PPARα activation, (KE 2) alteration of cell growth pathways, (KE 3) perturbation of cell growth and survival, and (KE 4) clonal expansion (Corton et al. 2014, 2018). It is well-established that *only* the first KE of this MOA (PPARα activation) is shared between humans and rodents due to key differences in the transcriptional networks controlled by rodent PPARα and human PPARα; PPARα-mediated gene expression in humans produces a subset (i.e. lipid modulating effects) of the responses observed in rodents (Corton et al. 2018; McMullen et al. 2020; Heintz et al. 2023). As such, liver effects in rodents associated with the downstream KEs of the PPARα MOA (e.g. cell proliferation, hepatocellular hypertrophy) are qualitatively not relevant for human health risk assessment. Despite the WOE supporting that HFPO-DA elicits liver toxicity in rodents via the PPARα MOA, other MOAs, including those involving PPARγ and cytotoxicity, have been hypothesized based on very limited evidence (USEPA 2021).

Recently, a set of in vitro transcriptomic studies was published that provide further support that the liver effects observed in rodents following HFPO-DA exposure occur via a PPARα MOA (Heintz et al. 2024a, 2024b). In these studies, primary hepatocytes from different species (i.e. mouse, rat, and pooled human) were treated with HFPO-DA or a number of prototypical agonists for the different hypothesized MOAs. These studies demonstrated highly concordant transcriptomic profiles between hepatocytes treated with HFPO-DA and hepatocytes treated with the prototypical PPARα agonist, GW7647, and a lack of concordance between HFPO-DA-treated hepatocytes and hepatocytes treated with the prototypical PPARγ agonist, rosiglitazone, or the prototypical cytotoxic agents, acetaminophen (APAP) or d-galactosamine. In addition, when treated with HFPO-DA or the PPARα or PPARγ agonists, a greater transcriptomic response was observed in rodent compared with human hepatocytes, indicating rodent hepatocytes are more sensitive to these chemicals (Heintz et al. 2024a). In addition, transcriptomic responses were also investigated in wild-type (WT) and *Ppara*-null mouse hepatocytes treated with HFPO-DA or a prototypical agonist for the different hypothesized MOAs. Transcriptomic profiles were concordant between WT or *Ppara*-null hepatocytes treated with HFPO-DA or GW7647. However, *Ppara*-null hepatocytes exhibited both a temporal and concentration-dependent delay in response to HFPO-DA or GW7647 treatment. This delay in transcriptomic response was not observed in *Ppara*-null hepatocytes treated with the prototypical agonists for PPARγ or cytotoxicity (Heintz et al. 2024b). Together, the findings from these 2 studies indicate a shared MOA between HFPO-DA and GW7647 in hepatocytes.

However, in vitro transcriptomic studies are limited in that they can only be used to examine the early KEs or the molecular initiating events of a chemical’s MOA. For example, regarding the PPARα MOA for HFPO-DA, the aforementioned in vitro studies primarily address KE 1, PPARα activation. Given the absence of nonparenchymal cells to facilitate latter KEs (i.e. those involving cell growth, proliferation, and survival), evaluation of downstream KEs of the PPARα MOA (e.g. KE 2: Alteration of cell growth pathways and KE 3: Perturbation of cell growth and survival) requires in vivo models. Specifically, proliferative responses from transient increases in DNA synthesis have been observed following acute (i.e. a few days) exposure to potent PPARα activators in rodent livers (Corton et al. 2018). Therefore, to further inform the MOA of HFPO-DA and evaluate the PPARα-dependence of HFPO-DA-mediated liver effects in vivo, phenotypic and transcriptomic responses in WT and *Ppara*-null mice were evaluated following short-term exposure to HFPO-DA or prototypical agonists with known MOAs (i.e. positive controls) for PPARα activation, PPARγ activation, or cytotoxicity (Fig. 1).

**Fig. 1.**
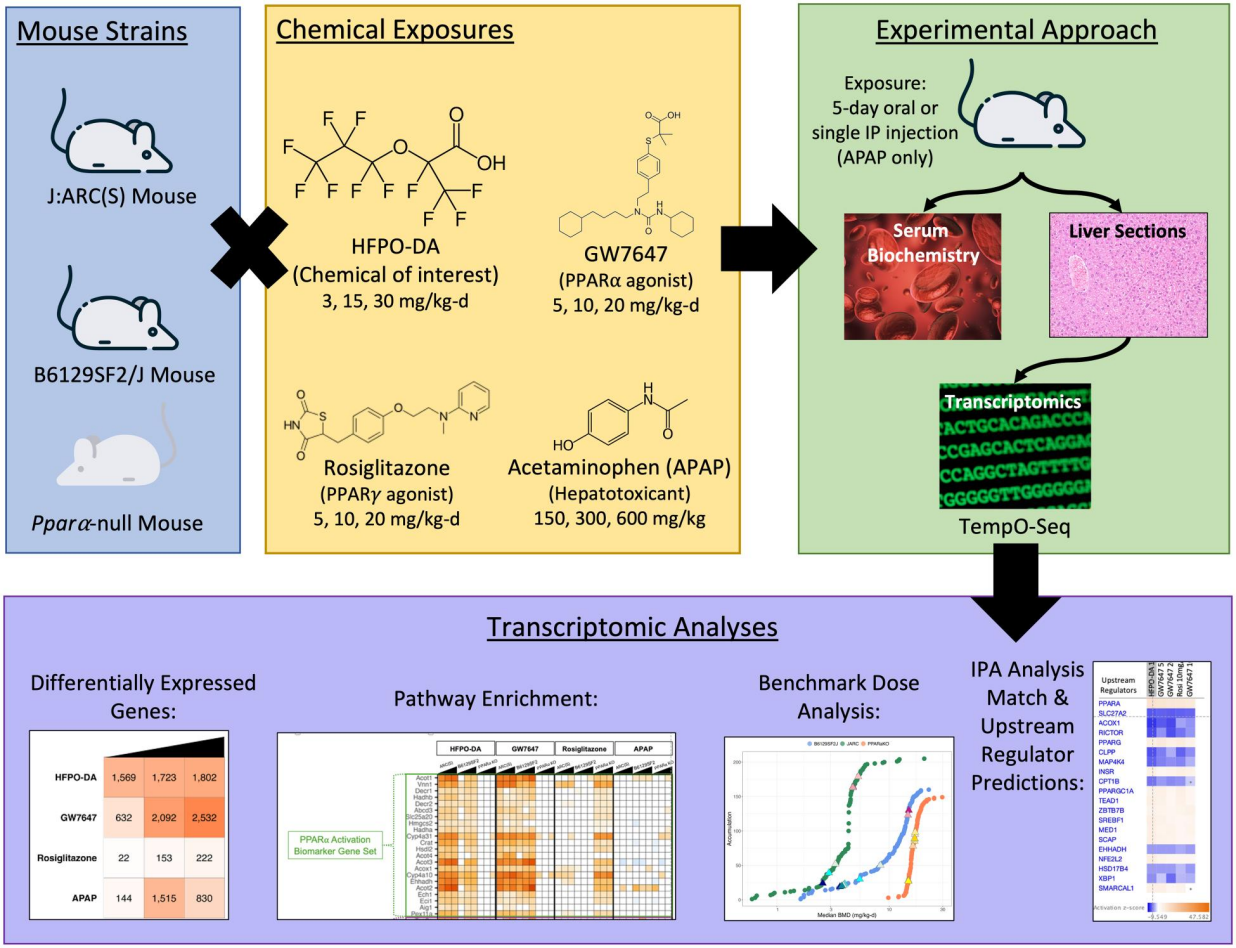
Experimental study design.

## Materials and methods

### Chemicals and dosing solutions

Ammonium perfluoro(2-methyl-3-oxahexanoate) (HFPO-DA; CASRN 62037-80-3; 95% purity) was purchased from Manchester Organics Ltd (Runcorn, Cheshire, United Kingdom). GW7647 (CASRN 265129-71-3; ≥98% purity) was purchased from Cayman Chemical Company (Ann Arbor, MI). Rosiglitazone (CASRN 122320-73-4; 98.9% purity), acetaminophen (APAP; CASRN 103-90-2; ≥99% purity), dimethyl sulfoxide (DMSO; CASRN 67-68-5; >99.9% purity), and carboxylmethylcellulose sodium salt (CASRN 9004-32-4; Product No. C4888) were purchased from Sigma Aldrich (St Louis, MO). HFPO-DA dosing solutions were prepared in deionized water; GW7647 and rosiglitazone dosing solutions were prepared in 5% DMSO suspended in 0.1% carboxymethylcellulose in deionized water. APAP dosing solutions were prepared in warm 0.9% saline.

### Animal husbandry and exposure

The study was conducted at The Jackson Laboratory (JAX) (Sacramento, CA) under the supervision and approval of the Institutional Animal Care and Use Committee (IACUC study No. 20110-SR) and under Good Laboratory Practices (GLP)-like conditions. Male mice, aged 9 to 12 wk, were acclimated for at least 2 d, and were housed in individually ventilated polysulfonate cages with HEPA-filtered air at a density of up to 5 mice per cage at a temperature of 20 °C to 26 °C and a relative humidity of 30% to 70%, under an ∼12-h light-dark cycle. Male mice were chosen because of their greater sensitivity than female mice to HFPO-DA-mediated liver effects as observed in previous studies (Thompson et al. 2019; Chappell et al. 2020; Heintz et al. 2022). Mice were provided filtered tap water, acidified to a pH of 2.5-3.0, and standard rodent chow ad libitum.

Three strains of mice were used, including 2 WT strains, J:ARC(S) (Stock No. 034608) and B6129SF2/J (Stock No. 101045), and the *Ppara*-null strain B6; 129S4-*Ppara^tm1Gonz^*/J (Stock No. 008154). J:ARC(S) mice, also referred to as JAX Swiss Outbred mice, are an outbred stock from Charles River Breeding Laboratories’ outbred CD-1(ICR) mouse colony. (Note: In 1981, mice from the CRL CD-1(ICR) colony were transferred to the Animal Resources Centre (ARC) in Australia. In 2020, this stock was transferred to JAX.) *Ppara*-null mice were generated by a targeted disruption of the ligand-binding domain of the mouse *Ppara* gene, rendering *Ppara* nonfunctional (see Lee et al. 1995 for details). *Ppara*-null mice are considered constitutive knockout mice (i.e. PPARα is nonfunctional in the entire animal). In order to be consistent with previous OECD guideline toxicity studies for HFPO-DA conducted in CD-1 mice, J:ARC(S) mice (JAX strain equivalent to CD-1 mice) were investigated in the current study. *Ppara*-null mice and their genetic background strain, B6129SF2/J mice, were used to further inform the MOA and PPARα-dependence of HFPO-DA-mediated liver effects. *Ppara*-null mice were generated in 129S4/SvJae mice by deletion of 83 base pairs in exon 8 of the ligand binding domain of the mPPARα gene, resulting in a nonfunctional gene (Lee et al. 1995). The lack of functional PPARα protein activity was verified through lack of activation of downstream PPARα target genes (Lee et al. 1995). No single genetic background strain of the *Ppara*-null mouse exists, but either the B6129SF2/J or C57BL/6J mouse strains are considered the most appropriate background strains.

This study consisted of 4 arms based on chemical treatment: HFPO-DA (chemical of interest), GW7647 (prototypical PPARα agonist), rosiglitazone (prototypical PPARγ agonist), and APAP (prototypical cytotoxic agent). The exposure route, dose level, and duration for HFPO-DA and each of the 3 prototypical agonists for the relevant hypothesized MOAs were designed to capture the transcriptomic signature and relevant phenotypic effects of each chemical’s MOA. Male mice from each of the 3 strains were randomly allocated based on body weight to each dose group (vehicle control, low, medium, or high) within each study arm (*n* = 4/dose/strain/arm) (Fig. S1).

Dosing solutions of HFPO-DA, GW7647, and rosiglitazone were administered via oral gavage at a dose volume of 10 ml/kg body weight once per day for 5 d. The doses were as follows: 0, 3, 15, or 30 mg/kg-d HFPO-DA; 0, 5, 10, or 20 mg/kg-d GW7647; and 0, 5, 10, or 20 mg/kg-d rosiglitazone. Dose levels for HFPO-DA were selected based on previous short-term and subchronic OECD test guideline toxicity studies in mice (DuPont 2008a, 2008b, 2010; Chappell et al. 2020). The highest dose, 30 mg/kg-d HFPO-DA was selected based on previous 7- and 28-d toxicity studies in mice; the lowest dose, 3 mg/kg-d HFPO-DA was selected based on minimal to no transcriptomic or histopathological changes following subchronic exposure to 0.1 or 0.5 mg/kg-d HFPO-DA. Dose levels for GW7647 and rosiglitazone were selected based on the mechanistic and physiological effects reported in previous mouse toxicity studies following short-term (5 to 9 d) exposure (Edvardsson et al. 1999; Shen et al. 2007; Tao et al. 2010; Foreman et al. 2021). The 5-d exposure duration used for HFPO-DA, GW7647, and rosiglitazone was selected to capture initial hepatic transcriptomic responses following chemical exposure. This duration is also consistent with the methods described in the EPA Transcriptomic Assessment Product (USEPA 2024).

APAP was administered to 12-h fasted mice as a single intraperitoneal (IP) injection at 0, 150, 300, or 600 mg/kg. Feed was restored immediately after APAP dosing and mice were sacrificed 6-h post-dose. Induction of acute APAP liver toxicity is both dose- and duration-sensitive (Bhushan et al. 2014); thus, in order to capture the transcriptomic signature for this mechanism, the experimental design employed was consistent with previous IP studies examining APAP-induced acute liver injury in mice (Bhushan et al. 2014; Kotulkar et al. 2023).

### Clinical observations and tissue isolation

Mice in the HFPO-DA, GW7647, and rosiglitazone study arms were monitored daily for clinical observations. Mice in the APAP arm were monitored closely after the single IP injection for severe adverse effects, including bleeding, severe lethargy, and blood in the urine: Low dose—single observation post-dosing, medium dose—observed once/hour for up to 6 h, and high dose—observations at 15-, 30-, 60-, 90-, and 120-min post-dose. If any severe adverse effects were observed in one or more animals within a dose group, all mice in that group were sacrificed *in extremis* at the same time. Mice that survived to scheduled study termination were euthanized via carbon dioxide asphyxiation either 24 h after receiving the last dose via oral gavage (HFPO-DA, GW7647, and rosiglitazone) or 6-h post-IP injection (APAP). Terminal body weights were taken prior to necropsy.

Immediately following sacrifice, blood and livers were collected. Blood was collected via cardiocentesis and processed to serum and shipped to IDEXX (West Sacramento, CA) for measurement of clinical chemistry parameters including alkaline phosphatase (ALP), aspartate aminotransferase (AST), alanine transaminase (ALT), total cholesterol, triglycerides, high-density lipoprotein, low-density lipoprotein, and total bile acids. Livers were weighed and fixed in 10% formalin prior to shipment to Experimental Pathology Laboratories, Inc. (EPL; Durham, NC), where the livers were embedded in paraffin (FFPE), sectioned sequentially at 4 to 6 µM, and mounted on glass slides.

### Histopathological examination

Liver sections were stained with hematoxylin and eosin (H&E) at EPL. The stained liver sections were evaluated by brightfield microscopy by an American College of Veterinary Pathologists (ACVP) board-certified veterinary pathologist and Fellow of the International Academy of Toxicologic Pathology (J.M.C.). The microscopic liver sections were evaluated and graded on a 5-point scale (1 to 5; minimal, mild, moderate, marked, severe) according to the scoring for focal and multifocal liver lesions by Thoolen et al. (2010). Hypertrophy was graded based on the proportion of hepatocytes affected: Minimal (1 to 3 hepatocytes affected around central vein), mild (4 to 8 hepatocytes affected), and moderate (most hepatocytes in lobule affected); higher scores are based on increased surface area of affected hepatocytes, but this was not observed in this study. In addition, the term “hypertrophy-atypical” was included to describe enlarged but vacuolated hepatocytes in the centrilobular region.

### Statistical analyses of phenotypic endpoints

Continuous datasets (e.g. body weight, liver weight, clinical pathology) were analyzed in GraphPad Prism (v10.4.0). Data were first assessed for homogeneity of group variances using the Bartlett’s test and for normality of residuals using the Shapiro–Wilk test. If the data passed both the Bartlett’s and Shapiro–Wilk tests, a 1-way analysis of variance *F*-test was used to assess differences among groups followed by a Dunnett’s test to compare each treatment group with concurrent controls. If the data failed one or both of the tests for variance and normality (i.e. *P*-value <0.05), then a nonparametric Kruskal–Wallis test was used to assess differences among groups followed by a Dunn’s test to compare each treatment group with concurrent controls.

### RNA preparation and sequencing

Mounted and unstained FFPE liver sections sequential to those used for histopathological examination were scraped from the slides and processed according to the TempO-Seq protocol by BioSpyder Technologies, Inc. (Carlsbad, CA) to yield libraries of tagged (by sample) detector oligos ligated to RNA targets, as previously described (Yeakley et al. 2017). These libraries were sequenced using a NovaSeq X Ultra-High-Throughput Sequencing System (Illumina, San Diego, California).

### Sequencing data processing and assessment of quality

Raw sequencing data for each sample (i.e. the FASTQ files) were analyzed using the TempO-Seq data analysis pipeline, as previously described (Yeakley et al. 2017). For each study arm, the output of this pipeline was a table containing the number of sequenced reads for each TempO-Seq probe per sample. Consistent with our previous studies, samples were reviewed for inclusion in the subsequent analyses based on 2 criteria: (i) overall sequencing depth ≥2 SDs below the mean across all samples within a study arm, and (ii) number of sequenced probes ≥2 SDs below the mean across all samples within a study arm (Chappell et al. 2020; Heintz et al. 2022, 2024a, 2024b). Samples that did not meet one or both criteria were excluded from subsequent analyses, which were conducted using packages in the R software environment (version 4.4.1; cran.r-project.org/).

### Differential gene expression analyses

For each study arm, count data were normalized with the DESeq2 R package (v1.44.0) (Love et al. 2014) to account for sample-to-sample variation in sequencing depth across samples. Statistical comparisons between treatment and control groups from the same mouse strain were performed in DESeq2 to identify differentially expressed probes (DEPs) and determine fold change of DEPs. DEPs were defined as probes with a false discovery rate (FDR) of <10% based on *P*-values adjusted for multiple comparisons using the Benjamini and Hochberg procedure (Love et al. 2014). In the TempO-Seq assay, some (but not all) genes are represented by multiple probes, such that the 30,146 mouse probes correspond to 21,398 mouse genes. Therefore, differentially expressed genes (DEGs) were identified from the corresponding DEPs (thus maintaining FDR <10%).

### Identification of pathway-level responses to exposure

Gene set enrichment analysis was used to identify biological pathways associated with transcriptomic responses in the livers of mice exposed to HFPO-DA or 1 of the 3 prototypical agonists for the hypothesized MOAs of interest. For genes with multiple corresponding probes, the probe with the highest baseMean (average normalized count value for a probe across all samples) within a study arm was used in the gene set enrichment analysis. In general, all probes for the same gene either met the criteria to be considered differentially expressed (FDR <10%) or did not meet the criteria; significant DEPs for the same gene had similar log fold change (logFC) values, thus, selection of the highest baseMean is not expected to alter biological interpretation of the data. To allow for comparison of enrichment results to previous in vitro transcriptomic studies (Heintz et al. 2024a, 2024b), mouse gene identifiers were converted into human identifiers (when available) with the R package biomaRt (v2.60.1) based on the Ensembl genome database (http://uswest.ensembl.org/index.html). Gene expression data with human identifiers were queried for enrichment of gene sets within the human canonical pathway (CP) subcollection (c2.cp.v2023.2). This collection includes gene sets from several pathway databases and is available through the Molecular Signatures Database (MSigDB; http://software.broadinstitute.org/gsea/msigdb/index.jsp). The hypergeometric test method for overrepresentation was used to identify enrichment of gene sets. DEGs (FDR <10%) for each treatment group within a study arm and mouse strain were tested for overrepresentation among gene sets in the CP subcollection using the Fisher combined probability test function within the Platform for Integrative Analysis of Omics data (PIANO) R package (v2.20.0) (Väremo et al. 2013). Gene sets with an FDR <5% were considered significantly enriched.

### Benchmark dose analyses

BMDExpress software (v2.3; Phillips et al. 2019) was used to conduct dose–response modeling from the normalized gene expression data from DESeq2. Data were loaded into BMDExpress without transformation, using TempO-Seq probe IDs as the gene identifiers. Genes altered by chemical exposure for each mouse strain were identified with a Williams trend test (*P*-value <0.05; absolute logFC ≥1.5 in at least 1 dose); corrections for multiple tests were not applied. Benchmark dose (BMD) analysis was conducted with the linear, power, hill, 2° and 3° polynomial, and exponential 2 to 5 models, assuming constant variance and a benchmark response of 1 SD. For each chemical and mouse strain, significant dose-responsive genes (DRGs) met the following criteria: A best BMD <10-fold below the lowest tested dose, a best BMD≤the highest tested dose, and a winning model fit *P*-value ≥0.1 (NTP 2018). The Reactome gene set collections available within the BMDExpress software were used for functional classification of significant DRGs and calculation of BMDs for enriched gene sets. Genes with BMD/BMD lower limit (BMDL) >20, BMD upper limit (BMDU)/BMD >20, and BMDU/BMDL >40 were removed from functional classification analyses (NTP 2018). There were no filters applied for minimum or maximum number of genes per gene set. Gene sets with a Fisher’s exact right *P*-value <0.05 were considered significantly enriched.

### Analysis match and upstream regulator predictions

Fold change and FDR values (<10%) determined by DESeq2 for DEGs were analyzed using Qiagen Ingenuity Pathway Analysis (IPA, v. 01-22-01; Qiagen Bioinformatics, Redwood City, California) software. For genes with multiple corresponding probes, the probe with the highest baseMean within a study arm was used. Gene expression profiles for HFPO-DA were compared and ranked against gene expression profiles of the prototypical agonist dose groups (excluding HFPO-DA) using IPA Analysis Match. The mid-dose group, 15 mg/kg-d HFPO-DA for each mouse strain was selected for analysis match comparisons to ensure that a maximum transcriptomic response was evaluated and avoid the potential for overt toxicity responses at the highest dose. In addition, gene expression profiles from the in vitro transcriptomic study in primary hepatocytes (Heintz et al. 2024a, 2024b) were also included in the analysis to evaluate the consistency between in vitro and in vivo transcriptomic responses.

Analysis matches were ranked using the overall experimental *z*-score. The overall *z*-score is a combined similarity score of the selected gene expression profile in question (i.e. mid-dose HFPO-DA for each mouse strain) compared with other transcriptomic profiles included in the analysis. The overall *z*-score is calculated using the average scores from IPA enrichment analysis of CP signatures, predicted upstream regulators, causal networks, and downstream effects. The top 3 analysis matches from prototypical agonist-exposed dose groups from the 5-d in vivo study herein, as well as the single top analysis match from previous in vitro transcriptomic studies (Heintz et al. 2024a, 2024b) were reported. For these top-ranked groups, activation/inhibition patterns of the top 20 predicted upstream regulators based on *z*-score (significance threshold >|2|) were examined.

## Results

### Phenotypic changes observed in WT and *Ppara*-null mice

All animals from the HFPO-DA, GW7647, and rosiglitazone study arms survived to scheduled necropsy. In the APAP arm, control and low-dose groups from all 3 mouse strains, as well as the mid-dose group from the J:ARC(S) strain, survived until scheduled necropsy (6-h post-injection). However, all other mid- and high-dose APAP groups were sacrificed early (between 1- and 3-h post-injection), *in extremis* based on severe lethargy (Supplementary File S1).

**Fig. 2.**
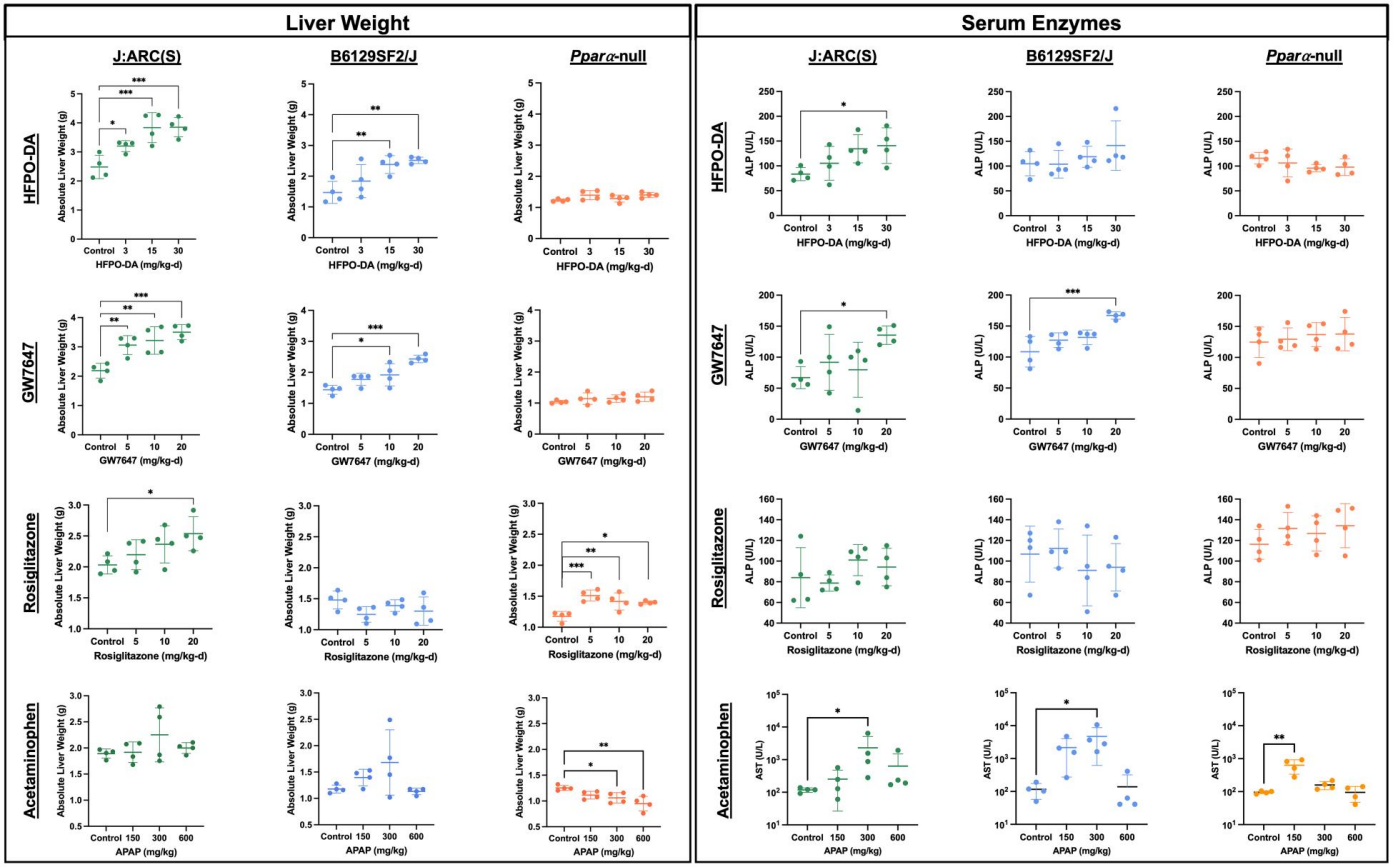
Liver weight and select serum liver enzyme changes in WT and *Ppara*-null mice exposed to HFPO-DA or a prototypical agonist for 5 d or 6 h (APAP only).

Body and organ weights collected from each study arm are summarized in Supplementary File S2. Within each mouse strain, terminal body weights did not differ significantly between dose groups for any of the chemicals tested. Liver weights were significantly increased in a dose-dependent manner in both WT mouse strains orally exposed to HFPO-DA or GW7647 for 5 d (Fig. 2). Liver weight was unaffected by HFPO-DA or GW7647 exposure in *Ppara*-null mice at any dose. Rosiglitazone caused a dose-dependent increase in liver weight in J:ARC(S) mice and a nonmonotonic elevation in liver weight in *Ppara*-null mice. Exposure to APAP did not cause significant changes in liver weight in WT mice but did cause a statistically significant decrease in *Ppara*-null mice at the higher doses despite early termination.

Clinical chemistry measurements for each study arm are summarized in Supplementary File S3. Compared with liver weight results, serum measurements, especially serum cholesterol and triglycerides, were more variable across treatment groups, likely due to the low replicate number (*N* = 4) and short exposure duration. Following exposure to HFPO-DA or GW7647, ALP, but not ALT or AST, was significantly increased in WT mice, whereas no serum liver enzymes were significantly altered in *Ppara*-null mice (Fig. 2). Exposure to rosiglitazone had no statistically significant effect on serum enzymes in any mouse strain. Following APAP exposure, ALT and AST (but not ALP) were significantly increased in WT mice at 300 mg/kg relative to controls; the lack of statistical significance at 600 mg/kg is likely due to early termination. In *Ppara*-null mice, exposure to 150 mg/kg APAP resulted in statistically significant increases in ALT and AST (Fig. 2), as well as nonsignificant increases in ALP (not shown). The lack of statistical significance at 300 and 600 mg/kg is likely due to early termination in these groups. Bile acids were not significantly altered in any mouse strain following exposure to HFPO-DA, GW7647, or rosiglitazone. In contrast, APAP significantly increased bile acids in WT mice at ≥300 mg/kg and in *Ppara*-null mice only at 300 mg/kg (Supplementary File S3).

**Fig. 3.**
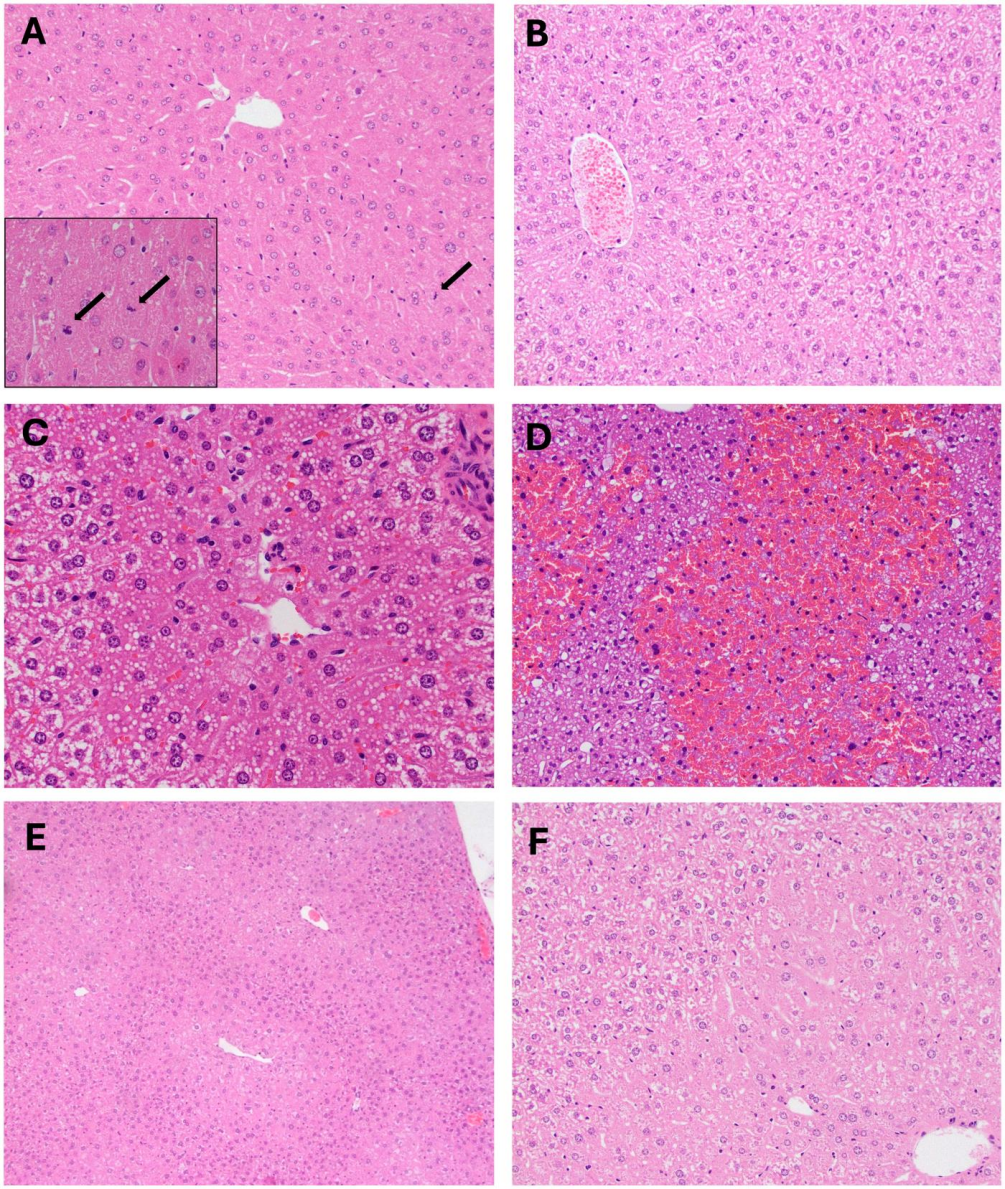
H&E-stained liver sections. Exposure to 30 mg/kg-d HFPO-DA for 5 d results in centrilobular hypertrophy and mitoses (arrows) in B6129SF2/J (A) and J:ARC(S) (A, inset) mice but not *Ppara*-null mice (B). Representative images of diffuse microvesicular steatosis in *Ppara*-null mice exposed to 150 mg/kg APAP (C) and acute centrilobular necrosis in B6129SF2/J mice exposed to 300 mg/kg APAP (D). Representative images of atypical hypertrophy in J:ARC(S) mice exposed to 20 mg/kg rosiglitazone at low (E) and high (F) magnification.

Consistent with the increased liver weight observed in WT mouse strains exposed to HFPO-DA or GW7647, the primary dose-related histological findings in these mice included hepatocellular hypertrophy, increased mitotic figures, and increased frequency of karyomegaly (enlarged nuclei from DNA replication) (Tables 1 and 2). Conversely, no hepatocellular hypertrophy or signs of cell proliferation were observed in *Ppara*-null mice (Fig. 3A and B). Extramedullary hematopoiesis was generally present in all exposure groups, albeit less so in B6129SF2/J mice. Broadly, exposure of WT mice to HFPO-DA or GW7647 may have reduced incidence of extramedullary hematopoiesis, although the sample sizes are too small to make definitive conclusions. A slight increase in the incidence of minimal extramedullary hematopoiesis in livers of *Ppara*-null mice exposed to HFPO-DA is likely an artifact of the uniquely low incidence (1/4) in control mice from this arm (Table 1).

**Table 1.** Summary of liver histopathological findings in mice^a^ orally exposed to HFPO-DA for 5 d.

	*Ppara*-null				B6129SF2/J				J:ARC (S)			
mg/kg/d	0	3	15	30	0	3	15	30	0	3	15	30
Rarefaction												
*Minimal*	2	1	4	4	0	0	1	3	1	4	4	0
*Mild*	2	3	0	0	2	4	0	0	2	0	0	0
*Moderate*	0	0	0	0	2	0	0	0	1	0	0	0
Infiltrate, mononuclear cell												
*Minimal*	0	2^c^	0	0	0	0	0	1^c^	0	0	0	1^c^
Extramedullary hematopoiesis												
*Minimal*	1	3	4	4	1	1	1	0	2	1	2	0
Karyomegaly												
*Minimal*	0	0	0	0	0	0	3	4	0	1	3	4
Pigment—present	0	0	0	0	0	0	0	0	0	0	0	1
Mitoses per 20× fields (range)^b^	0	0	0	0–1	0	0	0–9	0–7	0	0–1	0–5	1–10
Hypertrophy												
*Minimal*	0	0	0	0	0	3	0	1	0	2	0	0
*Mild*	0	0	0	0	0	1	2	1	0	2	3	1
*Moderate*	0	0	0	0	0	0	2	2	0	0	1	3

a *n* = 4 mice per group.

b Number of mitoses observed across all 4 mice per group.

c Indicates presence of apoptotic hepatocytes.

**Table 2.** Summary of liver histopathological findings in mice^a^ orally exposed to GW7647 for 5 d.

	*Ppara*-null				B6129SF2/J				J:ARC (S)			
mg/kg/d	0	5	10	20	0	5	10	20	0	5	10	20
Rarefaction												
*Minimal*	1	0	0	0	3	3	3	4	3	4	4	0
*Mild*	3	4	4	4	1	0	1	0	0	0	0	0
*Moderate*	0	0	0	0	0	0	0	0	0	0	0	0
Infiltrate, mononuclear cell												
*Minimal*	3^c^	1^c^	1^c^	2^c^	0	1	0	0	0	0	1^c^	0
Isolated apoptosis	0	0	0	0	1	0	0	0	0	0	0	0
Extramedullary hematopoiesis												
*Minimal*	4	3	3	4	1	0	0	0	0	1	2	1
Karyomegaly												
*Minimal*	0	0	0	0	0	0	2	3	1	0	2	3
Pigment—present	0	0	0	0	0	1	0	0	0	0	0	0
Mitoses per 20× fields (range)^b^	0	0	0	0	0	0–2	0–1	0–4	0	0–3	0	0–5
Hypertrophy												
*Minimal*	0	0	0	0	0	0	1	0	0	1	0	0
*Mild*	0	0	0	0	0	0	3	0	0	2	2	1
*Moderate*	0	0	0	0	0	0	0	4	0	0	2	3

a *n* = 4 mice per group.

b Number of mitoses observed across all 4 mice per group.

c Indicates presence of apoptotic hepatocytes.

The only notable histopathological changes in response to rosiglitazone exposure was atypical hypertrophy characterized by centrilobular hepatocytes that were larger than adjacent hepatocytes and had a distinct transition to smaller periportal hepatocytes (Fig. 3E and F; Table 3). However, the cytoplasm was at least minimally vacuolated suggesting that this was not typical enzyme induction. Notably, atypical hypertrophy was observed in the J:ARC(S) and *Ppara*-null mice, which both exhibited increased liver weight (Fig. 2).

**Table 3.** Summary of liver histopathological findings in mice^a^ orally exposed to rosiglitazone for 5 d.

	*Ppara*-null				B6129SF2/J				J:ARC (S)			
mg/kg/d	0	5	10	20	0	5	10	20	0	5	10	20
Rarefaction												
*Minimal*	0	0	0	0	0	2	2	3	3	1	3	2
*Mild*	4	4	4	4	4	2	2	1	0	3	1	2
*Moderate*	0	0	0	0	0	0	0	0	0	0	0	0
Infiltrate, mononuclear cell												
*Minimal*	1^b^	0	0	0	0	0	0	0	0	1^b^	2	0
Extramedullary hematopoiesis												
*Minimal*	4	1	4	4	0	1	1	3	1	0	2	2
Hypertrophy—atypical												
*Minimal*	0	0	0	2	0	0	0	0	0	2	3	4

a *n* = 4 mice per group.

b Indicates presence of apoptotic hepatocytes.

Exposure to APAP induced signs of cytotoxicity such as overt necrosis and microvesicular vacuolation (Fig. 3C and D), effects that were not observed in mice exposed to HFPO-DA, GW7647, or rosiglitazone. Compared with WT mice, necrosis appeared to be minimal in *Ppara*-null mice exposed to APAP, which might be explained by the short exposure duration in these mice due to early termination (see above). Either diffuse and/or centrilobular microvesicular vacuolation was observed in all mice exposed to APAP (Table 4).

**Table 4.** Summary of liver histopathological findings in mice^a^ 6-h post-IP injection of APAP.

	*Ppara*-null				B6129SF2/J				J:ARC (S)			
mg/kg/d	0	150	300	600	0	150	300	600	0	150	300	600
Rarefaction												
*Minimal*	0	0	0	0	2	3	4	1	4	2	0	1
*Mild*	4	4	0	0	2	1	0	0	0	2	1	0
Infiltrate, mononuclear cell												
*Minimal*	0	0	0	1	0	0	0	0	0	0	0	0
Infiltrate, mixed cell												
*Minimal*	0	3	0	0	0	0	0	0	0	1	0	0
Necrosis												
*Minimal*	0	1	0	0	0	0	3	0	0	0	0	0
*Mild*	0	0	0	0	0	2	0	0	0	1	0	0
*Moderate*	0	0	0	0	0	0	1	0	0	0	3	0
Microvesicular vacuolization, diffuse												
*Minimal*	0	0	0	0	0	2	0	0	0	0	0	0
*Mild*	0	0	1	3	0	0	4	0	0	0	1	2
*Moderate*	0	0	3	1	0	0	0	0	0	0	1	0
Microvesicular vacuolization, centrilobular												
*Minimal*	0	0	0	0	0	2	0	4	0	1	1	0
*Mild*	0	1	0	0	0	0	0	0	0	1	1	2
*Moderate*	0	3	0	0	0	0	0	0	0	0	0	0
Extramedullary hematopoiesis												
*Minimal*	4	4	2	2	1	0	1	3	3	3	1	1
Centrilobular glycogen depletion												
*Minimal*	0	0	0	0	0	0	0	0	0	1	0	0
*Mild*	0	0	0	0	0	0	1	0	0	2	2	0
*Moderate*	0	4	0	0	0	4	2	0	0	1	2	0

a *n* = 4 mice per group.

Overall, HFPO-DA caused phenotypic changes similar to the prototypical PPARα agonist GW7647 in WT mice including increased liver weight, hepatocellular hypertrophy, and increased hepatocellular proliferation. Additionally, similar changes in serum ALP were also observed in WT mice. The majority of these changes were not observed in *Ppara*-null mice exposed to HFPO-DA or GW7647. Increased liver weight in J:ARC(S) and *Ppara*-null mice exposed to the prototypical PPARγ agonist rosiglitazone was accompanied by atypical hypertrophy without signs of increased cell proliferation. APAP caused phenotypic effects not observed with HFPO-DA or GW7647 such as overt necrosis, microvesicular vacuolation, and increases in serum ALT and AST. These phenotypic differences observed with rosiglitazone and APAP demonstrate that HFPO-DA is not acting through a PPARγ or cytotoxicity MOA.

### Quantitative comparison of hepatic transcriptomic responses between HFPO-DA and prototypical agonists in WT and *Ppara*-null mice

A total of 6 samples were removed from the analysis across all chemical treatment groups and mouse strains (out of 192 samples total) following the assessment of sequencing data quality (criteria described in Materials and methods). In general, the samples removed were from different chemical treatment groups and mouse strains. Specific samples removed from downstream transcriptomic analyses are provided in Supplementary File S4.

The variance in transcriptomic profiles between each sample from a study arm was visualized using principal component analysis (PCA) (Fig. S2). Based on the separation between samples along PC1, dose and genotype explain much of the variance observed between the samples from mice exposed to HFPO-DA or GW7647; little separation was observed between samples from *Ppara*-null mice in PC1. In contrast, mouse strain appeared to largely contribute to the variance between samples from mice exposed to rosiglitazone in PC1. A batch effect was observed in PC2 for samples from HFPO-DA, GW7647, and rosiglitazone arms but not the APAP arm. (Note: The batch effect likely occurred during sequencing, as samples from HFPO-DA, GW7647, and rosiglitazone were sequenced together, whereas samples from the APAP study arm were sequenced separately.) Percent variance was almost equal between PC1 (28%) and PC2 (21%) for samples from the APAP study arm, with dose and mouse strain appearing to drive sample separation in PC1 and PC2, respectively.

**Fig. 4.**
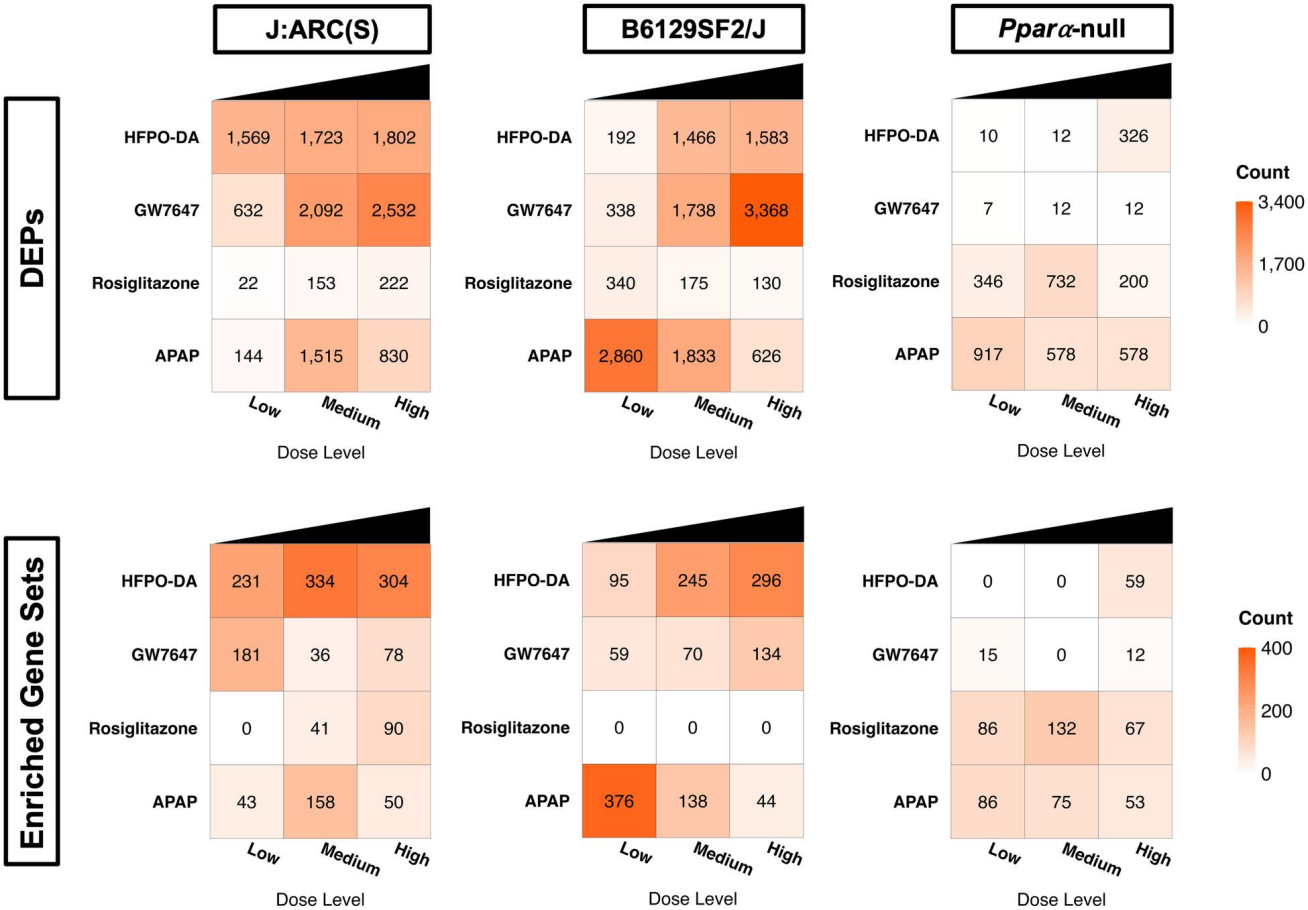
Number of significantly upregulated DEPs (relative to controls, FDR < 10%, no fold change filter) and enriched gene sets (FDR < 5%) in livers from mice exposed to HFPO-DA or a prototypical agonist chemical for 5 d or 6 h (APAP only). Each row represents a different chemical, and each column represents a different dose level, with doses increasing from left to right. For APAP-exposed mice, the number of DEPs and enriched gene sets were primarily related to timing of sacrifice (see Supplementary File S1) followed by dose level.

The number of significant (FDR <10% and no logFC filter) upregulated DEPs for each chemical dose group and mouse strain are presented in Fig. 4 (results for downregulated DEPs available in Fig. S3; also available in Supplementary File S5a–d). HFPO-DA or GW7647 exposure resulted in a substantially greater number of DEPs across dose groups in both WT mouse strains compared with *Ppara*-null mice. The low number of DEPs in *Ppara*-null mice exposed to HFPO-DA or GW7647 was consistent with the separation observed between samples from different mouse genotypes along PC1 of the PCA plots in Fig. S2. Conversely, a similar, or in some cases greater, number of DEPs across dose groups was observed in livers from rosiglitazone-exposed *Ppara*-null mice compared with WT mice. The number of DEPs in livers from APAP-treated mice was primarily related to timing of sacrifice (see Supplementary File S1) followed by dose level, with *Ppara*-null and B6129SF2/J mice being particularly sensitive to APAP treatment based on the premature sacrifice of both the mid- and high-dose groups in these strains.

The number of significant (FDR <5%) enriched gene sets using the hypergeometric test method also differed between mouse genotypes in HFPO-DA and GW7647-exposed dose groups, and to a lesser extent in rosiglitazone-exposed dose groups; enrichment was less affected by genotype in APAP-exposed dose groups (Fig. 4; downregulated gene set enrichment results in Fig. S3; also available in Supplementary File S6). The number of enriched gene sets across dose groups was considerably greater in livers from WT mouse strains compared with *Ppara*-null mice exposed to HFPO-DA or GW7647. No upregulated gene sets were significantly enriched in livers from the low- and mid-dose groups of HFPO-DA-exposed *Ppara*-null mice, and only a few gene sets were significantly enriched in GW7647-exposed *Ppara*-null mice across dose groups. In contrast, a substantially greater number of gene sets were enriched in the high-dose group of HFPO-DA-exposed *Ppara*-null mice. An opposite pattern was observed for rosiglitazone-exposed mice, where the number of enriched gene sets was generally greater in *Ppara*-null mice.

These data indicate a general lack of transcriptomic response in *Ppara*-null mice exposed to HFPO-DA or GW7647 based on the low number of DEPs. The minimal response at the gene level translated to little or no gene set enrichment in these dose groups of *Ppara*-null mice. Conversely, a substantially greater response at both the gene and pathway level was observed in WT mouse strains exposed to HFPO-DA or GW7647 and across mouse strains exposed to rosiglitazone and APAP.

### Comparison of gene set enrichment between HFPO-DA and prototypical agonists in WT and *Ppara*-null mice

At the pathway level, the types of enriched MSigDB gene sets were generally consistent across dose groups in WT mice exposed to HFPO-DA or GW7647 (Table 5; see Supplementary File S6 for B6129SF2/J gene set results), with gene sets related to fatty acid metabolism, cell cycle, DNA replication, and protein degradation among the top upregulated, and complement and coagulation cascade-related gene sets among the top downregulated for both chemicals. Regarding *Ppara*-null counterparts, enrichment of upregulated gene sets was only observed in the high-dose group for HFPO-DA, with enriched gene sets related to the upregulation of innate immune and inflammatory responses (Table S1). Conversely, the upregulated enriched gene sets in the low- and high-dose groups of GW7647-exposed *Ppara*-null mice were based on weak (i.e. logFC <2) induction of cytochrome P450 4a (*Cyp4a*) isoforms (see Discussion; Table S1; Supplementary Files S5 and S6). (Note: Prior to gene set enrichment analysis, differentially expressed Cyp4a genes in mice were converted to human homologs, CYP4A and CYP4F.) A weak, albeit consistent, downregulation of protease signaling was found in *Ppara*-null mice exposed to HFPO-DA, GW7647, or rosiglitazone. Fatty acid metabolism (but not cell cycle or protein metabolism) related gene sets were also among the top upregulated across dose groups for rosiglitazone in WT and *Ppara*-null mice. Across mouse strains exposed to APAP, commonly enriched upregulated gene sets included cell signaling cascades related to necroptosis, stress, and inflammation and downregulated gene sets consisted of cholesterol and steroid metabolism (Table 5; Table S1; Supplementary File S6).

**Table 5.** Top 5 most significantly enriched (up- and downregulated) gene sets determined by the hypergeometric test method in livers from J:ARC(S) mice.

Chemical	Upregulated Gene Set Name	**Adjusted** ***P*-value**	Downregulated Gene Set Name	**Adjusted** ***P*-value**
HFPO-DA	***3 mg/kg/d***			
	REACTOME Respiratory Electron Transport ATP Synthesis by Chemiosmotic Coupling and Heat Production by Uncoupling Proteins	1.28E-21	REACTOME Alpha Defensins	1.64E-03
	REACTOME Citric Acid TCA Cycle and Respiratory Electron Transport	2.15E-21	–	–
	WP Electron Transport Chain Oxphos System in Mitochondria	1.09E-20	–	–
	REACTOME Respiratory Electron Transport	2.35E-17	–	–
	REACTOME Fatty Acid Metabolism	2.12E-16	–	–
	***15 mg/kg/d***			
	REACTOME Fatty Acid Metabolism	1.30E-22	KEGG Complement and Coagulation Cascades	1.86E-14
	KEGG MEDICUS Variant Mutation Inactivated UBQLN2 to 26S Proteasome Mediated Protein Degradation	7.64E-21	WP Complement Activation	1.11E-09
	KEGG MEDICUS Variant Mutation Inactivated VCP to 26S Proteasome Mediated Protein Degradation	7.64E-21	BIOCARTA Complement Pathway	2.37E-09
	REACTOME The Role of GTSE1 in G2 M Progression After G2 Checkpoint	7.64E-21	WP Complement and Coagulation Cascades	2.37E-09
	REACTOME Synthesis of DNA	9.63E-21	BIOCARTA Lectin Pathway	5.69E-07
	***30 mg/kg/d***			
	REACTOME Fatty Acid Metabolism	4.52E-21	KEGG Complement and Coagulation Cascades	8.89E-08
	REACTOME Protein Localization	1.83E-16	WP Complement Activation	1.83E-06
	KEGG Peroxisome	2.05E-16	BIOCARTA Complement Pathway	4.31E-06
	WP Fatty Acid Beta Oxidation	5.28E-15	REACTOME Drug ADME	1.15E-05
	REACTOME Cell Cycle Checkpoints/REACTOME M Phase	1.28E-13	WP Complement and Coagulation Cascades	2.99E-05
GW7647	***5 mg/kg/d***			
	REACTOME Fatty Acid Metabolism	8.49E-26	BIOCARTA Complement Pathway	2.93 E-03
	WP Fatty Acid Beta Oxidation	1.14E-17	BIOCARTA Lectin Pathway	2.93E-03
	KEGG PPAR Signaling Pathway	2.23E-17	WP Complement Activation	2.93E-03
	KEGG Fatty Acid Metabolism	5.94E-17	KEGG MEDICUS Reference Common Pathway of Complement Cascade MAC Formation	3.56E-03
	WP PPAR Signaling Pathway	1.63E-15	BIOCARTA Classic Pathway	3.98E-03
	***10 mg/kg/d***			
	REACTOME Eukaryotic Translation Elongation	9.10E-30	KEGG Complement and Coagulation Cascades	2.78E-17
	KEGG MEDICUS Reference Translation Initiation	6.97E-29	WP Complement and Coagulation Cascades	5.43E-14
	KEGG Ribosome	7.99E-28	REACTOME Regulation of Insulin-like Growth Factor (IGF) Transport and Uptake by Insulin-like Growth Factor Binding Proteins (IGFBPS)	5.09E-08
	WP Cytoplasmic Ribosomal Proteins	7.99E-28	WP Complement Activation	9.34E-07
	REACTOME Response of EIF2AK4 GCN2 to Amino Acid Deficiency	8.40E-27	WP Complement System	9.34E-07
	***20 mg/kg/d***			
	REACTOME Eukaryotic Translation Elongation	8.32E-31	KEGG Complement and Coagulation Cascades	4.59E-17
	KEGG Ribosome	4.95E-30	WP Complement and Coagulation Cascades	8.21E-14
	KEGG MEDICUS Reference Translation Initiation	1.39E-29	REACTOME Metabolism of Amino Acids and Derivatives	2.16E-08
	WP Cytoplasmic Ribosomal Proteins	5.09E-28	WP Urea Cycle and Associated Pathways	2.40E-08
	REACTOME Response of EIF2AK4 GCN2 to Amino Acid Deficiency	1.87E-26	WP Metabolism Epileptic Disorders	3.29E-08
Rosiglitazone	***5 mg/kg/d***			
	–	–	–	–
	–	–	–	–
	–	–	–	–
	–	–	–	–
	–	–	–	–
	***10 mg/kg/d***			
	KEGG PPAR Signaling Pathway	1.61E-13	–	–
	WP PPAR Signaling Pathway	2.12E-12	–	–
	KEGG Fatty Acid Metabolism	4.91E-07	–	–
	REACTOME Fatty Acid Metabolism	4.91E-07	–	–
	WP Eicosanoid Metabolism via Lipoxygenases LOX	6.37E-07	–	–
	***20 mg/kg/d***			
	KEGG PPAR Signaling Pathway	3.60E-19	BIOCARTA PEPI Pathway	9.44E-03
	WP PPAR Signaling Pathway	1.26E-19	–	–
	REACTOME Fatty Acid Metabolism	1.27E-15	–	–
	KEGG Peroxisome	1.82E-06	–	–
	WP Eicosanoid Metabolism via Cyclooxygenases COX	1.82E-06	–	–
Acetaminophen	***150 mg/kg***			
	WP NRF2 Pathway	4.96E-08	–	–
	WP Nuclear Receptors Meta Pathway	7.57E-08	–	–
	REACTOME HSF1 Dependent Transactivation	1.97E-07	–	–
	PID ATF2 Pathway	2.15E-07	–	–
	REACTOME Attenuation Phase	4.72E-07	–	–
	***300 mg/kg***			
	KEGG MEDICUS Reference Type II Interferon to JAK STAT Signaling Pathway	1.39E-12	–	–
	KEGG MEDICUS Pathogen HIV TAT to TLR2,4 NFKB Signaling Pathway	2.02E-10	–	–
	KEGG MEDICUS Reference IFN RIPK1/3 Signaling Pathway	6.15E-10	–	–
	KEGG MEDICUS Reference RIG I NFKB Signaling Pathway	1.42E-09	–	–
	KEGG MEDICUS Reference TLR7,9 IRF7 Signaling Pathway	3.70E-09	–	–
	***600 mg/kg***			
	KEGG MEDICUS Reference cGAS-STING Signaling Pathway	6.23E-13	WP Cholesterol Biosynthesis Pathway	5.17E-08
	KEGG MEDICUS Pathogen HIV TAT to TLR2,4 NFKB Signaling Pathway	5.76E-12	REACTOME Activation of Gene Expression by SREBF (SREBP)	5.73E-06
	KEGG MEDICUS Reference RIG I NFKB Signaling Pathway	1.85E-11	WP Cholesterol Synthesis Disorders	5.73E-06
	KEGG MEDICUS Reference TLR3 IRF7 Signaling Pathway	3.03E-11	REACTOME Cholesterol Biosynthesis	9.31E-06
	KEGG MEDICUS Reference TLR7,9 IRF7 Signaling Pathway	6.66E-11	WP Cholesterol Metabolism with Bloch and Kandutsch-Russell Pathways	1.07E-05

No significantly enriched gene set (adjusted *P*-value <0.05).

**Fig. 5.**
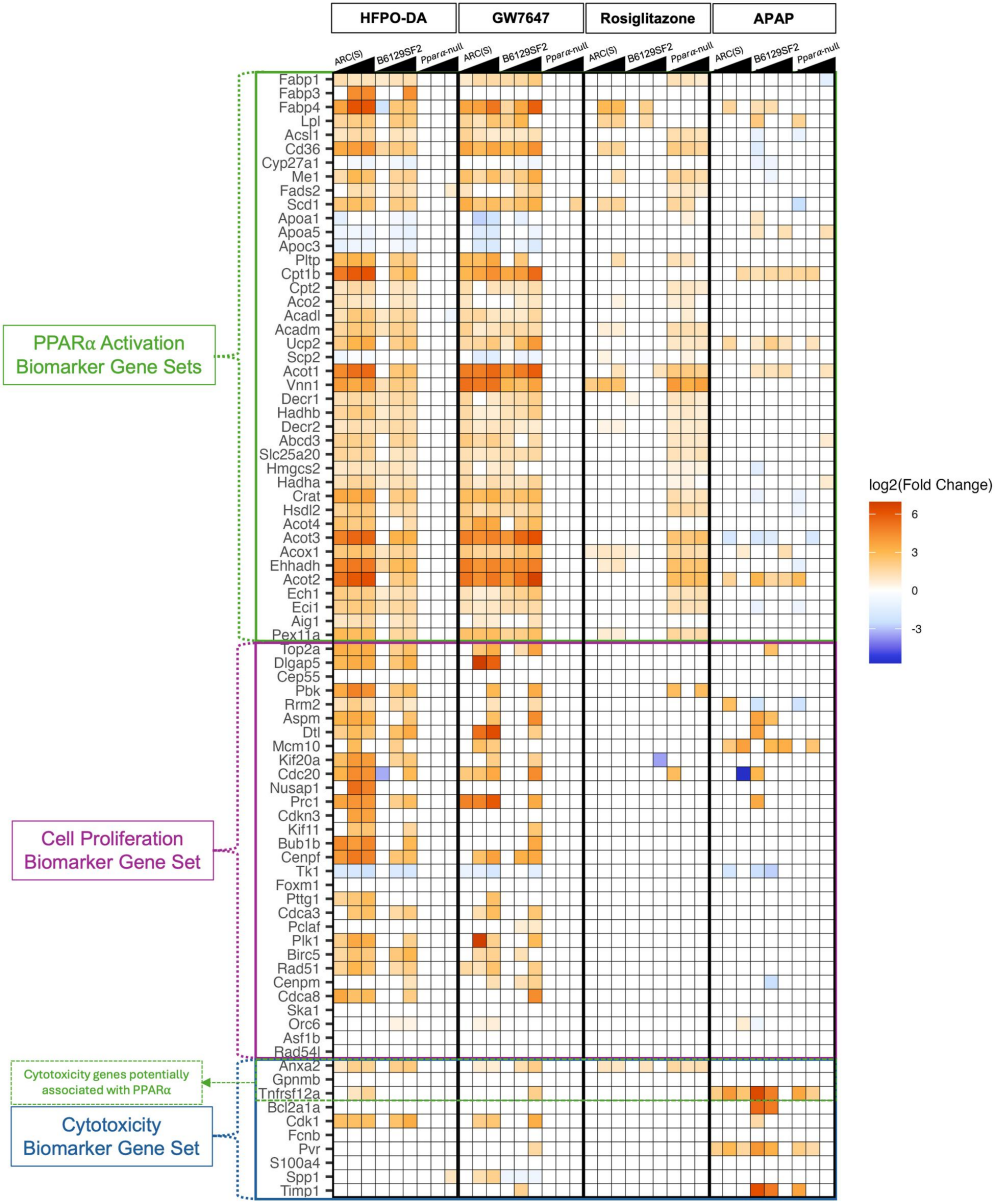
Heatmap of hepatic gene expression for PPARα activation, cell proliferation, and cytotoxicity biomarkers in mice exposed to HFPO-DA or a prototypical agonist chemical. Significant (FDR < 10%) DEGs (rows) are indicated by orange (upregulated) or blue (downregulated) colors; intensity of colors is based on the fold change value for the gene within a dose group, mouse strain, and chemical exposure (columns). White cells indicate that a gene was not significantly altered for a chemical dose group following exposure compared with respective controls. Right triangles at the top of the heatmap indicate increasing dose levels from left to right for each chemical and mouse strain. Note: The PPARα activation biomarker is based on data from rats and mice (Podtelezhnikov et al. 2020; KEGG Pathway: Mmu03320). The cytotoxicity biomarker was developed using rats (Corton et al. 2020) and the cell proliferation biomarker was developed using mice, rats, and human cells (Corton et al. 2024).

In addition to the gene sets available through the MSigDB, gene expression biomarkers for PPARα activation (Podtelezhnikov et al. 2020; PPARα target genes within the Kyoto Encyclopedia of Genes and Genomes [KEGG] PPAR Signaling Pathway for Mus musculus [mmu03320; https://www.genome.jp/dbget-bin/www_bget?mmu03320]), cell proliferation (Corton et al. 2024), and cytotoxicity (Corton et al. 2020) were also evaluated across the chemical dose groups and mouse strains tested (Fig. 5). A consistent increase in the expression of genes that are part of the PPARα activation *and* cell proliferation biomarkers was observed in HFPO-DA or GW7647 WT mouse strains; this pattern was not observed in rosiglitazone or acetaminophen-exposed WT mice. Compared with HFPO-DA and GW7647, rosiglitazone induced low expression of the PPARα activation biomarker gene sets in WT mouse strains, but uniquely increased expression of the biomarker in *Ppara*-null mice, albeit without concomitant biomarkers for cell proliferation. The few cytotoxicity biomarker genes that were increased in WT mice (but not *Ppara*-null mice) exposed to HFPO-DA or GW7647, namely *Anxa2*, *Tnfrsf12a*, and *Cdk1*, have also been associated with PPARα activity (*Anxa2* and *Tnfrsf12a*) (Oshida et al. 2015; Heintz et al. 2023) and cell proliferation (*Cdk1*) (Liu et al. 2008). These genes were likely increased in response to the proliferation burst observed in WT mouse livers exposed to HFPO-DA or GW7647. In contrast, cytotoxicity biomarker genes were consistently increased across mouse strains in APAP-treated mice, with a relatively higher fold change compared with the genes increased in HFPO-DA, GW7647, and rosiglitazone-exposed mice. The lack of significant changes in cytotoxicity biomarker gene expression in the high-dose groups for APAP-treated B6129SF2/J and *Ppara*-null mice is likely a result of the early sacrifice of these groups. Overall, these data indicate pathway-level responses between HFPO-DA and the prototypical PPARα agonist, GW7647, were consistent and genotype-specific.

### Comparison of transcriptomic dose-responses between HFPO-DA and prototypical agonists in WT and *Ppara*-null mice

**Fig. 6.**
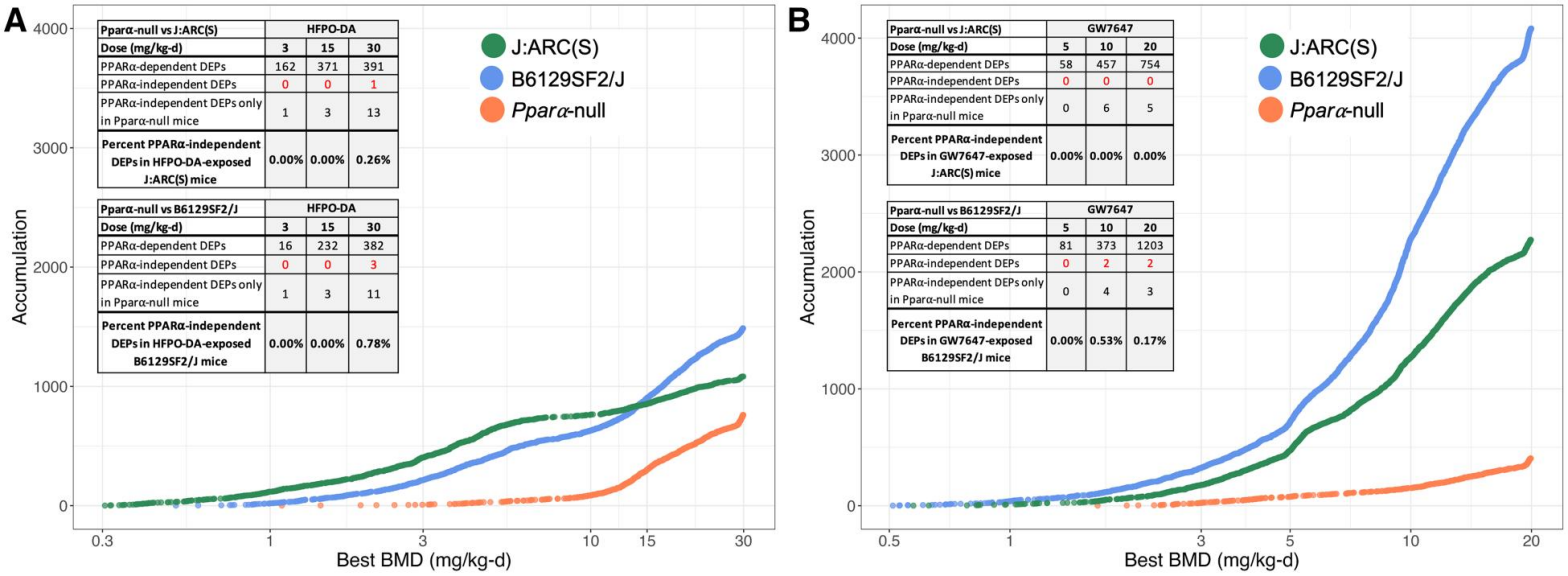
Accumulation plots of best BMDs among significant dose-responsive probes (best-fit *P*-value ≥0.1; best BMD <10-fold below the lowest dose, best BMD≤highest dose; BMD/BMDL <20; BMDU/BMD <20; BMDU/BMDL <40) in livers from J:ARC(S) (green points), B6129SF2/J (blue points), and *Ppara*-null (orange points) mice orally exposed to HFPO-DA (A) or GW7647 (B) for 5 d. Inset tables present the percent of PPARα-independent DEPs (FDR < 5%; |logFC| ≥1.5) for each dose group in WT mouse strains exposed to HFPO-DA (A) or GW7647 (B). The percent PPARα-independent DEPs within each WT mouse strain exposed to HFPO-DA or GW7647 were calculated using the approach described in Fig. 1 of Rosen et al. (2017). DEPs within each dose group that were not significantly dose-responsive were removed prior to determining the percent PPARα-independent DEPs, thereby eliminating DEPs not directly related to chemical exposure.

**Fig. 7.**
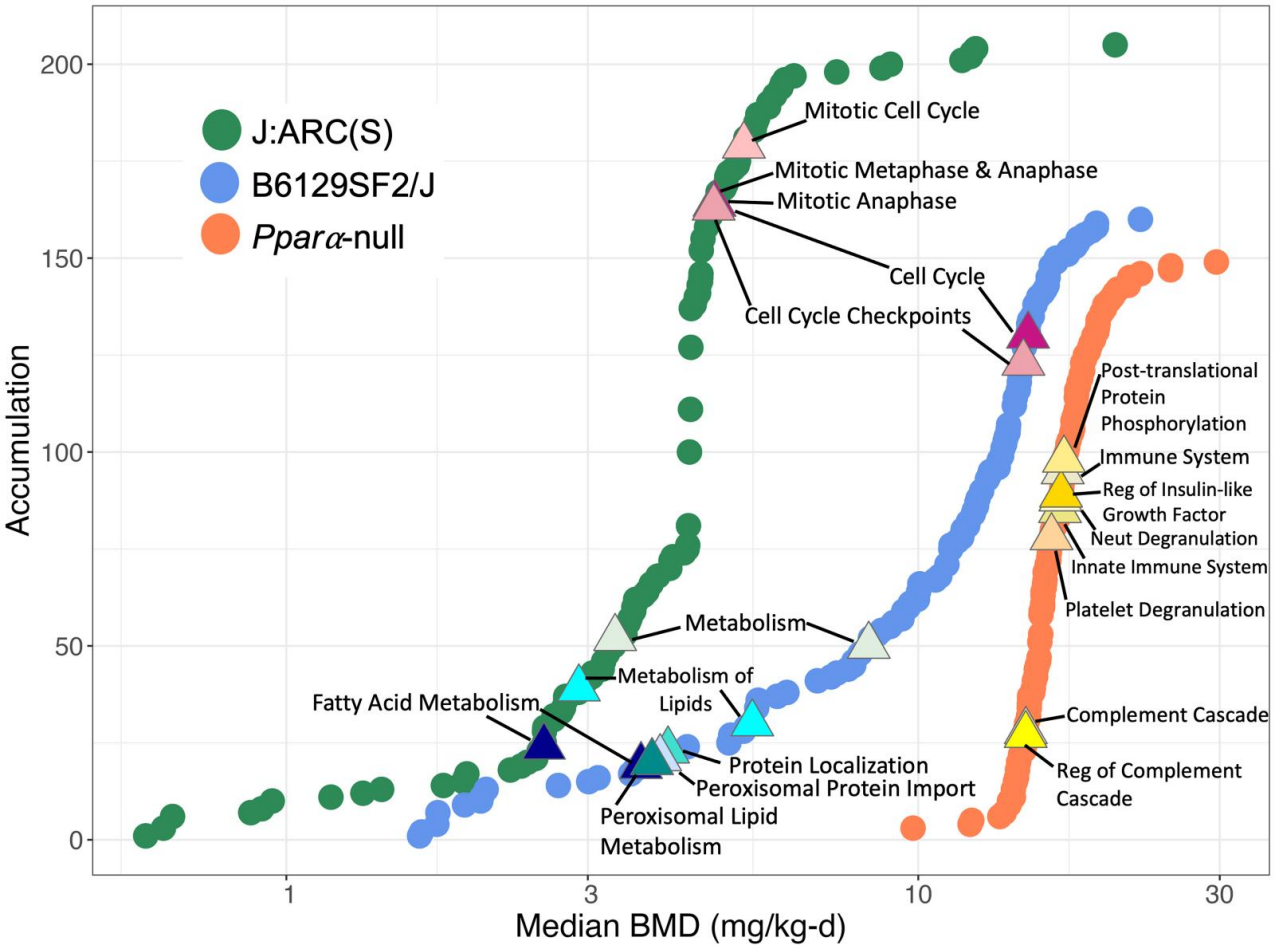
Accumulation plots of median BMDs for significantly enriched pathways (Fisher’s exact right *P*-value <0.05) among DRGs in livers from HFPO-DA-exposed J:ARC(S) (green points), B6129SF2/J (blue points), and *Ppara*-null (orange points) mouse strains. The top 8 most significantly enriched signaling pathways (based on Fisher’s exact right *P*-value) in each mouse strain are annotated by color-coded triangles. All of the top-most enriched pathways highlighted in this figure had an overall upregulated direction of transcriptional regulation.

BMD modeling of transcriptomic responses also reflected the consistent, and genotype-specific, responses in HFPO-DA and GW7647-exposed mice (Figs 6 and 7; Supplementary Files S7 and S8). Compared with WT mice, a substantially lower number of probes were dose-responsive in *Ppara*-null mice exposed to HFPO-DA or GW7647, particularly at dose levels <10 mg/kg-d (Fig. 6). Using DEPs and significant dose-responsive probe results, the percent of PPARα-independent DEPs within each WT mouse strain exposed to HFPO-DA or GW7647 were calculated for each dose group using the approach described in Rosen et al. (2017). Briefly, DEPs (FDR < 5%; |logFC| ≥1.5) were divided into 3 classes: (i) PPARα-dependent in WT mice; (ii) PPARα-independent and differentially expressed in both genotypes; (iii) PPARα-independent and differentially expressed only in *Ppara*-null mice. Percent of PPARα-independent DEPs was calculated by dividing the number of DEPs in class ii by the sum of DEPs in classes i and ii. DEPs within each dose group that were not dose-responsive were removed prior to determining the percent of PPARα-independent DEPs, thereby eliminating DEPs not directly related to chemical exposure. (Note: Dose-response modeling was not performed by Rosen et al. (2017) (possibly because only 1-2 dose groups tested).) As shown in the tables inset in Fig. 6, less than 1% of DEPs across all dose groups in either WT mouse strain are PPARα-independent following HFPO-DA or GW7647 exposure. These findings indicate that the upregulated inflammatory/innate immune responses observed in the high dose group (30 mg/kg-d) of *Ppara*-null mice exposed to HFPO-DA are idiosyncratic to *Ppara*-null mice and not relevant to HFPO-DA-exposed WT mice. The results for GW7647 are comparable to those reported by Rosen et al. (2017) following short-term exposure to another prototypical PPARα agonist, WY-14,643 (2% PPARα-independent at 50 mg/kg-d). However, the results for HFPO-DA differ from the results for the other PFAS investigated in Rosen et al. (2017), such as perfluorooctanoic acid (PFOA; 14% PPARα-independent at 3 mg/kg-d).

Consistent with the hypergeometric gene set enrichment results by dose group (see above; Table 5), the top significantly enriched signaling pathways among DRGs were also related peroxisome lipid metabolism, fatty acid metabolism, mitosis, cell cycle, and DNA replication in HFPO-DA-exposed WT mice and a high-dose-specific upregulation of inflammatory and innate immune response signaling in *Ppara*-null counterparts (Fig. 7 and Supplementary File S8). Median BMDs for significantly enriched signaling pathways among DRGs were up to 8-fold higher in livers from HFPO-DA-exposed *Ppara*-null mice compared with WT counterparts. Specifically, median BMDs for enriched signaling pathways were between ∼15 to 30 mg/kg-d HFPO-DA in *Ppara*-null mice compared with ∼1.8 to 15 mg/kg-d HFPO-DA in WT mice. Combined with the gene set enrichment results described above (Fig. 5; Table S1; Supplementary File S6), findings for HFPO-DA-exposed *Ppara*-null mice suggest that exposure to 30 mg/kg-d HFPO-DA elicited off-target transcriptomic responses that were unrelated to cell proliferation, cytotoxicity, or PPARα-specific genes and pathways. Importantly, these off-target, high-dose transcriptomic responses were not accompanied by changes in liver weight, histopathology, or clinical chemistry. In general, median BMDs of signaling pathways related to peroxisome activity or beta-oxidation of fatty acids were around the lowest dose tested in both WT mouse strains, i.e. 3 mg/kg-d HFPO-DA, whereas median BMDs for pathways related to mitosis and cell cycle were around 5 mg/kg-d HFPO-DA for J: ARC(S) and 15 mg/kg-d HFPO-DA for B6129SF2/J mice (Fig. 7).

In GW7647-exposed mice, only WT mouse strains had significant enrichment of signaling pathways among DRGs (Supplementary File S8). The types of enriched signaling pathways among DRGs in GW7647-exposed WT mice were similar to that of HFPO-DA-exposed counterparts, with enrichment of signaling pathways related to mitosis, cell cycle, and protein synthesis occurring at median BMDs of ∼10 mg/kg-d GW7647 in both WT mouse strains (Supplementary File S8). However, DRGs underlying pathways related to fatty acid metabolism were largely filtered out based on the criteria described in the Materials and methods section for low BMDs. When some of the filtering criteria were removed (e.g. BMD/BMDL >20, BMDU/BMDL >40, BMD value >10-fold below lowest dose), significant enrichment of signaling pathways related to peroxisomal lipid metabolism was observed in GW7647-exposed WT mice, with median BMDs ∼1 mg/kg-d GW7674 (data not shown).

For rosiglitazone, due to the lower number of significant DRGs across mouse strains (Supplementary File S7), there was no significant enrichment of signaling pathways in rosiglitazone-exposed WT or *Ppara*-null mice. Dose-response modeling of gene expression data from APAP-exposed mice was not performed due to differences in exposure duration resulting from variable timing of premature sacrifice across APAP dose groups.

### Assessment of similarity and upstream regulation of gene expression profiles from WT and *Ppara*-null mice exposed to HFPO-DA or prototypical agonists

**Fig. 8.**
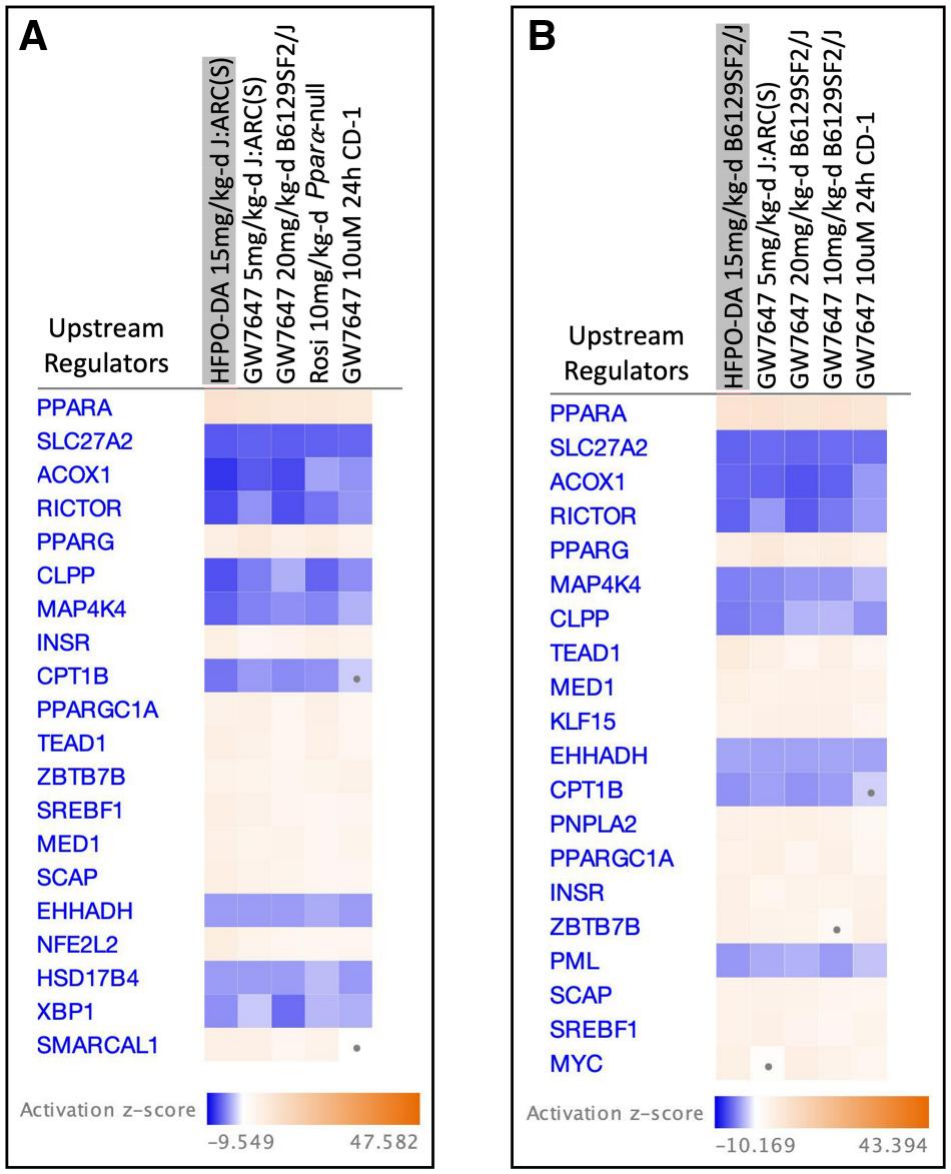
Chemical dose groups with the highest overall similarity *z*-score to J:ARC(S) (A) and B6129SF2/J (B) mice exposed to 15 mg/kg-d HFPO-DA (grey highlight) using IPA Analysis Match. Each subsequent column after 15 mg/kg-d HFPO-DA shows the top 3 analysis matches from the 5-d in vivo study herein in addition to the single top analysis match from previous in vitro transcriptomic studies (Heintz et al. 2024a, 2024b). The overall *z*-score is calculated using the average scores from IPA enrichment analysis of CP signatures, predicted upstream regulators, causal networks, and downstream effects. Activation (orange color) and inhibition (blue color) patterns of the top 20 predicted upstream regulators based on *z*-score are shown in the heatmaps; intensity of each color increases with the absolute *z*-score. A square containing a dot indicates the *z*-score did not meet the significance threshold of >|2|.

Qiagen IPA Analysis Match was used to compare and rank hepatic gene expression profiles from mice exposed to 15 mg/kg-d HFPO-DA to profiles for the prototypical agonists to identify which non-HFPO-DA groups had the highest overall similarity *z*-score. Gene expression profiles of GW7647-exposed WT (both J:ARC(S) and B6129SF2/J strains) mice were ranked as having the highest overall similarity *z*-score to profiles of HFPO-DA-exposed WT mouse strains (Fig. 8). Interestingly, the transcriptomic profile of *Ppara*-null mice exposed to 10 mg/kg-d rosiglitazone was the third highest ranked profile in similarity to HFPO-DA-exposed J:ARC(S) mice (Fig. 8A); these findings concur with the observed induction of the PPARα biomarker gene set in rosiglitazone-exposed *Ppara*-null (compared with the weak expression in WT) mice as described above (see Fig. 5). In addition, to evaluate the consistency between in vitro and in vivo transcriptomic responses, hepatic gene expression profiles from 15 mg/kg-d HFPO-DA-exposed WT strains were compared with transcriptomic profiles from the previous transcriptomic studies in primary hepatocytes (Heintz et al. 2024a, 2024b). The top-ranked in vitro transcriptomic profile was CD-1 strain (WT) mouse hepatocytes exposed to GW7647 for 24 h (Fig. 8). No significant matches (i.e. overall *z*-scores >|2|) were identified for *Ppara*-null mice exposed to 15 mg/kg-d HFPO-DA due to the lack of DEGs.

When the IPA Analysis Match was reversed, and the hepatic transcriptomic profiles of mice exposed to 10 mg/kg-d GW7647 were examined for similarity to other chemical dose groups, profiles from HFPO-DA-exposed WT mouse groups (both in vivo and in vitro) ranked the highest for overall similarity to GW7647-exposed WT mouse strains (Fig. S4); similar to HFPO-DA, no significant matches were identified for *Ppara*-null mice exposed to 10 mg/kg-d GW7647.

In addition, gene expression profiles of the top-ranked groups from the IPA analysis matches for HFPO-DA or GW7647-exposed WT mice showed similar activation/inhibition patterns of predicted upstream regulators across the top-ranked groups, with PPARα consistently predicted as the top upstream regulator (based on *z*-score) (Fig. 8; Fig. S4).

## Discussion

This 5-d in vivo study is an extension of previous in vitro work comparing the hepatic responses of HFPO-DA to hepatic responses from prototypical agonists for the hypothesized MOAs of interest, i.e. PPARα, PPARγ, and cytotoxicity MOAs (Heintz et al. 2024a, 2024b). Consistent with that previous in vitro work, hepatic responses following short-term in vivo exposure to HFPO-DA were highly concordant with the prototypical PPARα agonist GW7647 but distinct from the prototypical PPARγ agonist rosiglitazone and cytotoxic agent APAP. A major disparity between the in vivo and in vitro studies is that HFPO-DA and GW7647 elicited a delayed transcriptomic response in *Ppara*-null hepatocytes in vitro that was not recapitulated in vivo. In fact, a general lack of transcriptomic and phenotypic changes was observed following in vivo exposure to HFPO-DA or GW7647 in *Ppara*-null mice. The delayed temporal response in vitro was posited to be the result of toxicokinetic factors of the test systems (Heintz et al. 2024b). Responses in *Ppara*-null hepatocytes were weaker, yet consistent with the types of transcriptomic signaling pathways (e.g. fatty acid metabolism and PPAR signaling) enriched in WT mouse hepatocytes, suggesting evidence for compensatory mechanisms activated in the absence of PPARα and/or as a consequence of the inability to pharmacokinetically eliminate these chemicals from the in vitro test system (Heintz et al. 2024b). Conversely, HFPO-DA and GW7647 were likely rapidly eliminated following in vivo exposure based on the elimination half-life of HFPO-DA and other PPARα agonists like GW7647 (Chapman 1987; Gannon et al. 2016). The lack of responses in *Ppara*-null mice exposed to these chemicals indicates that the hepatic effects observed in WT counterparts are >99% PPARα-dependent. Broadly, some of the disparities in in vivo and in vitro responses highlight the continued need for mechanistic research in animal models.

Upregulated transcriptomic responses unique to *Ppara*-null mice exposed to 30 mg/kg-d HFPO-DA were limited to enrichment of inflammatory signaling pathways. However, these mice exhibited none of the phenotypic changes in liver weight, histopathology, or clinical chemistry observed in WT mice—suggesting that the gene changes are off-target and high-dose-specific. Indeed, application of a logFC filter of |logFC| ≥1.5 decreases the total number of significant (FDR < 10%) DEPs in *Ppara*-null mice exposed to 30 mg/kg-d HFPO-DA from 466 to 83, indicating that most of the gene expression changes observed in this group were marginal and are unlikely relevant to WT mice; this is further supported by the minimal overlap in PPARα-independent DEPs between WT and *Ppara*-null mice. The limited response in *Ppara*-null mice in this study is consistent with previous studies that also reported an absence of hepatic gene expression changes in *Ppara*-null mice exposed to lower doses of HFPO-DA over longer exposure durations, i.e. 0.3 mg/kg-d in drinking water for 20 wk with a high-fat diet (Attema et al. 2022) and 0.5 mg/kg-d via oral gavage for 28 d (Ren et al. 2024).

*Ppara*-null mice exposed to the prototypical PPARα agonist GW7647 exhibited no significant gene set enrichment among DRGs (i.e. based on BMD modeling in BMDExpress); however, a few enriched gene sets were upregulated in the low- and high-dose groups of *Ppara*-null mice. DEGs underlying this enrichment consisted solely of *Cyp4a* isoforms; however, the magnitude of *Cyp4a* induction in these *Ppara*-null mice was weak (logFC values ranging from 1.2 to 2.0) compared with WT mice exposed to GW7647 (logFC values ranging from 4 to 7). Although *Cyp4a* is commonly thought of as a specific target of PPARα activation, previous studies have also reported induction of *Cyp4a* subfamily members by several PPARα activators in both WT and *Ppara*-null mice (Ren et al. 2009; Oshida et al. 2015). In addition to PPARα, GW7647 can also activate other PPAR subtypes including PPARγ, albeit with a 1000-fold lower potency compared with that of PPARα (Brown et al. 2001). Given the competing roles of energy homeostasis in the liver between PPARα and PPARγ (Wang et al. 2020), it is possible that in lieu of PPARα, PPARγ may play a larger role in the regulation of hepatic gene expression despite its lower expression level in the liver (Chawla et al. 1994). Apart from PPARs, hormones such as androgens, estrogens, and growth hormone have also been reported to play a regulatory role in *Cyp4a* activation in mouse liver and kidney (Zhang and Klaassen 2013); therefore, the weak activation of *Cyp4a* in GW7647-exposed *Ppara*-null mice may be explained, at least in part, by PPARγ and/or hormonal regulation. Importantly, these nominal transcriptomic findings were not accompanied by phenotypic changes.

In contrast to the overall lack of phenotypic and transcriptomic changes in *Ppara*-null mice exposed to HFPO-DA or GW7647, exposure to the prototypical PPARγ agonist rosiglitazone or cytotoxic agent APAP elicited similar, if not greater phenotypic and transcriptomic responses in *Ppara*-null mice compared with WT mice. These observations indicate that the responses observed from exposure to these prototypical agonist chemicals are not PPARα-dependent and are mediated through different MOAs than HFPO-DA and GW7647. Across rosiglitazone-exposed mouse strains, enrichment of gene sets related to PPAR signaling and fatty acid metabolism were observed at each dose level, however, these signaling pathways were not dose-responsive in any mouse strain. Within each dose group, a more robust enrichment of fatty acid metabolism signaling was observed in livers from rosiglitazone-exposed *Ppara*-null mice relative to WT counterparts. This greater enrichment of fatty acid metabolism-related pathways in *Ppara*-null mice was also observed in rosiglitazone-treated *Ppara*-null hepatocytes from our previous in vitro transcriptomic studies (Heintz et al. 2024b). As noted above, PPARα and PPARγ have competing roles for maintaining energy homeostasis in the liver, thus in the absence of PPARα, it is predicted that PPARα target genes can be regulated through other mechanisms, such as PPARγ. Of note, the activation of PPARα target genes in rosiglitazone-exposed *Ppara*-null mice was not accompanied by an increase in cell proliferation signaling or histopathological increases in mitotic figures or karyomegaly, as observed in WT mice exposed to HFPO-DA or GW7647, further supporting a different MOA for rosiglitazone-mediated responses.

*Ppara*-null mice were also more sensitive to APAP exposure, based on overt toxicity observations resulting in early sacrifice. WT and *Ppara*-null mice that survived to scheduled study termination exhibited effects consistent with early liver cytotoxicity, including overt necrosis, microvesicular vacuolation, increases in serum ALT and AST, as well as upregulation of cell signaling cascades associated with necroptosis, inflammation, and stress. Previous studies investigating the role of PPARα in APAP-induced hepatotoxicity have observed hepato-protective effects from PPARα activation via PPARα agonist treatment, whereas this protective effect was lost in WT mice only treated with APAP or *Ppara*-null mice (Chen et al. 2000; Patterson et al. 2012). Conversely, APAP-induced liver injury was significantly reduced in hepatocyte-specific *Ppara* knockout mice, indicating that PPARα expression in other cell types, e.g. nonparenchymal cells, may mediate APAP’s hepatoxic mechanisms (Zhang et al. 2022). Thus, findings for APAP-exposed mice from the current study are generally consistent with previous studies indicating a slightly/potentially protective role of PPARα in APAP-induced hepatotoxicity (Chen et al. 2000; Patterson et al. 2012).

The phenotypic and transcriptomic findings in WT mice following short-term exposure to HFPO-DA were consistent with findings from previous subchronic toxicity and transcriptomic studies for HFPO-DA (Chappell et al. 2020; Heintz et al. 2022) and add to the WOE supporting the PPARα MOA for HFPO-DA-mediated liver effects in mice. Specifically, data for HFPO-DA from the current study inform the early KEs (KEs 1 to 3) of the PPARα MOA including: (KE 1) dose-dependent increases in hepatocellular hypertrophy, decreased serum lipids, and enrichment of PPARα, fatty acid metabolism, and peroxisomal lipid metabolism signaling across dose levels; (KE 2) dose-related increases in transcriptomic signaling of mitosis, DNA replication, and cell cycle; and (KE 3) dose-dependent increases in liver weight, mitotic figures, and frequency of karyomegaly. The latter transcriptomic and histopathological findings for KE 2 and 3 are consistent with the proliferative responses from transient increases in DNA synthesis and are expected following short-term exposure to potent PPARα activators (Corton et al. 2018).

In addition to hepatocellular proliferation, PPARα-mediated liver growth in rodents can also involve suppression of apoptosis. Evidence for apoptosis suppression by PPARα activators has primarily been measured in in vitro studies whereas the low baseline levels of apoptosis in vivo precludes detection of suppression (Corton et al. 2018). Thus, findings for decreased apoptosis may be an artifact of the in vitro test systems used. For HFPO-DA, previous in vitro studies also found decreased apoptotic signaling in both mouse and rat hepatocytes (Heintz et al. 2024a). In the current in vivo study, weak transcriptomic signaling (i.e. mixed direction in transcriptomic regulation) for apoptosis was observed in HFPO-DA-exposed WT mice, with no apparent changes in apoptosis in H&E-stained liver sections. In contrast, increased apoptosis has been reported in rodent studies following longer exposures (i.e. >90 d) to PPARα activators or HFPO-DA (Marsman et al. 1992; Chappell et al. 2020; Thompson et al. 2025). This increase in apoptosis following sustained activation of PPARα is likely a homeostatic response to counterbalance hepatomegaly resulting from increased hepatocellular proliferation and hypertrophy.

Aside from the phenotypic findings discussed above in support of the PPARα MOA for HFPO-DA-exposed WT mice, the serum liver enzyme, ALP, was also uniquely increased in HFPO-DA and GW7647-exposed WT mice, whereas increased serum ALT and AST were observed in APAP-exposed WT and *Ppara*-null mice. Increased serum ALP has been proposed as a biomarker for cholestasis, hepatic microsomal enzyme induction, peroxisomal proliferation, and hepatocellular hypertrophy, with no evidence as a marker for necrosis, whereas serum ALT and AST are biomarkers of hepatocellular injury (Ennulat et al. 2010; Hall et al. 2012; Pollock and Minuk 2017). As discussed in Heintz et al. (2023), PPARα activators and PFAS can disrupt bile acid homeostasis in a PPARα-dependent manner via perturbation of hepatic bile acid transporter gene expression (Cheng and Klaassen 2008; Gyamfi and Wan 2009; Liu et al. 2014; Xie et al. 2019). Gene expression results herein also show PPARα-dependent downregulation of basolateral transporters, *Slc10a* (Na^+^-dependent bile acid transporter, *Ntcp*) and *Slco1a* (organic anion transporting polypeptide 1a1, *Oatp1a1*) (see Supplementary File S5), in HFPO-DA and GW7647-exposed mice. Although these transcriptomic findings were not accompanied by changes in serum bile acids, increased serum bile acids have been measured in mice following longer exposure to HFPO-DA or other established PPARα agonists (Gyamfi and Wan 2009; DuPont 2010). Thus, while not directly related to the KEs of the PPARα MOA, the genotype-specific increases in serum ALP provide further support for a PPARα-mediated response in mice following HFPO-DA exposure.

Some PFAS, including HFPO-DA, have been hypothesized to inhibit the highly conserved orphan nuclear receptor hepatocyte nuclear factor 4 alpha (HNF4α) and consequently upregulate genes putatively repressed by HNF4α, e.g. cell cycle genes *Ccna2*, *Ccnb1*, *Ccnd1*, *Ccne1*, and *Cdk4* in studies using human hepatocytes (Walesky et al. 2013; Beggs et al. 2016; Robarts et al. 2022). Based on differential gene expression results herein, these HNF4α target genes are activated by both HFPO-DA and GW7647 in WT but not *Ppara*-null mice, illustrating that the upregulation of these genes is PPARα dependent (see Supplementary File S5). Previous studies with the established PPARα agonist WY-14,643 found decreased HNF4α protein levels in WT but not *Ppara*-null mouse livers, with no changes in HNF4α mRNA levels, indicating that PPARα activators post-transcriptionally downregulate HNF4α protein levels through PPARα (Shin et al. 2006). A similar decrease in HNF4α protein was also found in human hepatocytes treated with WY-14,643 (Marrapodi and Chiang 2000). Altogether, these studies in rodents (in vivo) and human hepatocytes (in vitro) suggest that PPARα activators, including some PFAS (e.g. HFPO-DA), may indirectly inhibit HNF4α by activating PPARα. HNF4α and PPARα also regulate the expression of amino acid catabolism genes; PPARα and its heterodimer partner, retinoid X receptor alpha, were recently found to downregulate hepatic amino acid catabolism in mice by promoting the proteasomal degradation of HNF4α (Tobón-Cornejo et al. 2021). In accordance with these findings, enrichment of gene sets related to protein metabolism and degradation was observed in HFPO-DA and GW7647-exposed mice in a PPARα-dependent manner. HFPO-DA and other PFAS have also been speculated to downregulate expression of the glucocorticoid receptor (GR) gene and GR-mediated signaling pathways in human cells and rodents, respectively (Zhang et al. 2023; Jackson et al. 2024). This inhibition of GR signaling by PFAS is also likely an indirect effect of PPARα activation, as glucocorticoids can activate PPARα (Lemberger et al. 1994) and both synergistic and antagonistic effects have been observed between PPARa and GR following co-treatment with established agonists of each receptor (Bougarne et al. 2009).

In summary, the 5-d in vivo study herein builds upon previous in vitro transcriptomic studies in rodent and human hepatocytes to investigate PPARα-dependent and independent mechanisms of HFPO-DA. Results from previous in vitro transcriptomic studies in primary hepatocytes were inherently limited as they only address KE 1, PPARα activation, given the absence of nonparenchymal cells to facilitate downstream KEs of the PPARα MOA (i.e. those involving cell growth, proliferation, and survival). In vivo models with intact liver cell populations inform KEs 1 to 3 of the PPARα MOA leading from nuclear receptor activation to altered cell growth and survival resulting in phenotypic changes associated with hepatomegaly. Combined, these data do not support hypotheses that HFPO-DA is acting through either a cytotoxic or PPARγ MOA to elicit the liver effects observed in rodent studies (USEPA 2021). The consistent phenotypic and transcriptomic signaling patterns between HFPO-DA and the prototypical PPARα agonist (GW7647) in WT mice, and the lack of changes in *Ppara*-null mice, provide further support that the KEs downstream of PPARα activation (alteration of cell growth, perturbation of cell cycle, and clonal expansion) observed in mice following exposure to HFPO-DA are PPARα-dependent and occur via the PPARα MOA for rodent liver tumors. Given the wide consensus that the KEs downstream of PPARα activation in this MOA lack human relevance due to key differences in transcriptional networks controlled by rodent PPARα and human PPARα (Corton et al. 2018; McMullen et al. 2020; Heintz et al. 2023), effects in the mouse liver should not serve as the basis of toxicity values for the purposes of human health risk assessment of HFPO-DA.

## Supplementary Material

kfaf049_Supplementary_Data

## Data Availability

RNA sequencing data are publicly available at NCBI’s Gene Expression Omnibus (https://www.ncbi.nlm.nih.gov/geo/) (GEO series accession number: GSE282998). Supplementary Files (S4-S8) are publicly available at Dryad Digital Repository (https://datadryad.org/) (DOI: 10.5061/dryad.hx3ffbgq5).
