# Supplementary material for: Comparison of phenotypic and transcriptomic profiles between HFPO-DA and prototypical PPARα, PPARγ, and cytotoxic agents in wild-type and Ppara-null mouse livers
Source: Toxicol Sci. 2025 Apr 11;206(1):183–201. doi: 10.1093/toxsci/kfaf049 (PMC12198672; doi:10.1093/toxsci/kfaf049)
Supplement: kfaf049_Supplementary_Data [file kfaf049_supplementary_data.zip › kfaf049_Supplementary_Data/toxsci-24-0611-File010.pdf]

Supplementary Tables

Table S1. Top 5 most significantly enriched (up- and downregulated) gene sets determined by the hypergeometric test method in livers from *Ppar*α-null mice.

Chemical	Upregulated Gene Set Name	Adjusted *p*-value	Downregulated Gene Set Name	Adjusted *p*-value
HFPO-DA	***3 mg/kg/day***			
	-	-	REACTOME Alpha Defensins	6.69E-17
	-	-	REACTOME Defensins	7.21E-10
	-	-	REACTOME Antimicrobial Peptides	7.36E-08
	-	-	BIOCARTA Pepi Pathway	1.73E-06
	-	-	REACTOME Uptake of Dietary Cobalamins into Enterocytes	4.13E-06
	15 mg/kg/day			
	-	-	-	-
	-	-	-	-
	-	-	-	-
	-	-	-	-
	-	-	-	-
	30 mg/kg/day			
	KEGG Complement and Coagulation Cascades	6.69E-10	BIOCARTA Pepi Pathway	0.01297316
	WP Complement and Coagulation Cascades	6.69E-10	REACTOME Activation of Matrix Metalloproteinases	0.04153316
	REACTOME Response to Elevated Platelet Cytosolic Ca2+	5.96E-09	REACTOME Cobalamin (Cbl, vitamin B12) Transport and Metabolism	0.01297316
	REACTOME Regulation of Insulin-like Growth Factor (IGF) transport and uptake by Insulin-like Growth Factor Binding Proteins (IGFBPs)	1.67E-07	REACTOME Digestion of Dietary Lipid	0.01297316
	WP Complement System	2.73E-07	REACTOME Uptake of Dietary Cobalamins into Enterocytes	0.0011921
GW7647	***5 mg/kg/day***			
	REACTOME Eicosanoids	2.05E-08	REACTOME Alpha Defensins	9.95E-18
	REACTOME Fatty Acids	2.83E-08	REACTOME Antimicrobial Peptides	1.13E-08
	WP Eicosanoid Metabolism via Cytochrome P450 Monooxygenases Pathway	5.20E-06	REACTOME Defensins	1.09E-10
	REACTOME Cytochrome P450 Arranged by Substrate Type	5.23E-06	-	-
	WP Oxidation by Cytochrome P450	5.23E-06	-	-
	10 mg/kg/day			
	-	-	-	-
	-	-	-	-
	-	-	-	-
	-	-	-	-
	-	-	-	-
	20 mg/kg/day			
	REACTOME Eicosanoids	2.86E-07	REACTOME Alpha Defensins	2.60E-12
	REACTOME Fatty Acids	3.94E-07	REACTOME Defensins	1.54E-07

	WP Eicosanoid Metabolism via Cytochrome P450 Monooxygenases Pathway	3.12E-05	REACTOME Antimicrobial Peptides	4.30E-06
	REACTOME Cytochrome P450 Arranged by Substrate Type	6.01E-05	REACTOME Digestion of Dietary Lipid	0.03725999
	REACTOME Miscellaneous Substrates	6.01E-05	-	-
Rosiglitazone	***5 mg/kg/day***			
	REACTOME Fatty Acid Metabolism	2.32E-25	REACTOME Alpha Defensins	2.02E-13
	KEGG Peroxisome	2.46E-15	REACTOME Defensins	1.89E-06
	REACTOME Protein Localization	1.53E-14	BIOCARTA Pepi Pathway	0.00010495
	KEGG PPAR Signaling Pathway	7.04E-13	REACTOME Antimicrobial Peptides	0.00012488
	KEGG Fatty Acid Metabolism	7.79E-13	REACTOME Uptake of Dietary Cobalamins into Enterocytes	0.00018718
	10 mg/kg/day			
	REACTOME Fatty Acid Metabolism	5.67E-22	-	-
	REACTOME Citric Acid TCA Cycle & Respiratory Electron Transport	1.07E-16	-	-
	REACTOME Protein Localization	1.56E-16	-	-
	KEGG Fatty Acid Metabolism	2.65E-15	-	-
	REACTOME Mitochondrial Fatty Acid Beta Oxidation	2.65E-15	-	-
	20 mg/kg/day			
	REACTOME Fatty Acid Metabolism	3.86E-29	REACTOME Uptake of Dietary Cobalamins into Enterocytes	2.35E-05
	KEGG PPAR Signaling Pathway	2.11E-15	REACTOME Activation of Matrix Metalloproteinases	2.75E-05
	WP PPAR Signaling Pathway	2.64E-14	REACTOME Cobalamin (Cbl, vitamin B12) Transport and Metabolism	0.00026704
	REACTOME Peroxisomal Protein Import	2.17E-13	BIOCARTA PEPI Pathway	0.0007035
	KEGG Peroxisome	2.18E-13	REACTOME Metabolism of Vitamins and Cofactors	0.00472716
Acetaminophen	***150 mg/kg***			
	KEGG MAPK Signaling Pathway	1.74E-06	WP Cholesterol Metabolism with Bloch and Kandutsch-Russell Pathways	8.29E-10
	REACTOME Nuclear Events Kinase & Transcription Factor Activation	1.74E-06	WP Cholesterol Biosynthesis Pathway	2.73E-09
	WP MAPK Signaling Pathway	6.73E-06	REACTOME Cholesterol Biosynthesis	6.91E-09
	REACTOME NGF Simulated Transcription	6.97E-06	WP Cholesterol Synthesis Disorders	1.84E-08
	REACTOME Attenuation Phase	1.11E-05	REACTOME Activation of Gene Expression by SREBF (SREBP)	1.21E-07
	300 mg/kg			
	KEGG MEDICUS Reference TLR3/IRF7 Signaling Pathway	2.32E-14	REACTOME Activation of Gene Expression by SREBF (SREBP)	1.01E-11
	KEGG MEDICUS Pathogen HIV TAT to TLR2,4 NFKB Signaling Pathway	8.13E-14	REACTOME Regulation of Cholesterol Biosynthesis SREBF (SREBP)	8.77E-10
	KEGG MEDICUS Reference cGAS-STING Signaling Pathway	1.90E-13	WP Cholesterol Synthesis Disorders	8.77E-10

	KEGG MEDICUS Reference TLR7,9 IRF7 Signaling Pathway	1.22E-12	WP Cholesterol Biosynthesis Pathway	2.14E-09
	KEGG MEDICUS Reference Type II Interferon to JAK STAT Signaling Pathway	2.48E-12	WP Cholesterol Metabolism with Bloch and Kandutsch-Russell Pathways	5.28E-09
	600 mg/kg			
	REACTOME Estrogen Dependent Gene Expression	0.00142956	WP Cholesterol Biosynthesis Pathway	2.85E-17
	REACTOME Diseases of Programmed Cell Death	0.00365344	WP Cholesterol Synthesis Disorders	2.67E-15
	REACTOME FOXO Mediated Transcription	0.00365344	REACTOME Activation of Gene Expression by SREBF (SREBP)	3.39E-15
	REACTOME Oxidative Stress induced Senescence	0.00365344	REACTOME Cholesterol Biosynthesis	4.09E-15
	REACTOME Metabolism of Amino Acids and Derivatives	0.0043576	REACTOME Regulation of Cholesterol Biosynthesis SREBF (SREBP)	8.85E-13

Key: - : no significantly enriched gene set (adjusted *p* value <0.05)
