# Supplementary material for: Comparison of phenotypic and transcriptomic profiles between HFPO-DA and prototypical PPARα, PPARγ, and cytotoxic agents in wild-type and Ppara-null mouse livers
Source: Toxicol Sci. 2025 Apr 11;206(1):183–201. doi: 10.1093/toxsci/kfaf049 (PMC12198672; doi:10.1093/toxsci/kfaf049)
Supplement: kfaf049_Supplementary_Data [file kfaf049_supplementary_data.zip › kfaf049_Supplementary_Data/toxsci-24-0611-File011.pdf]

Supplementary Figures

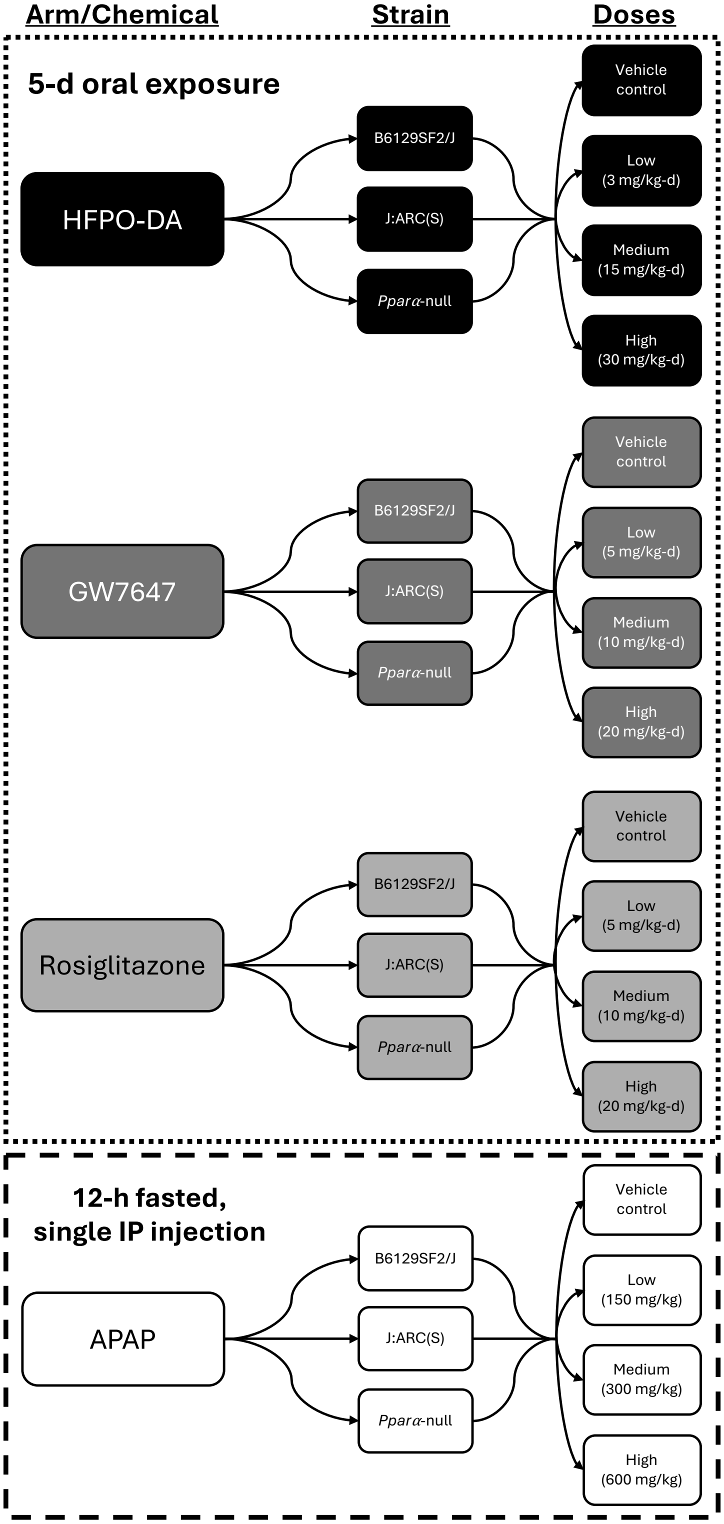

Supplementary Figure S1. Mouse strains and doses tested within each of the four experimental arms of the study.

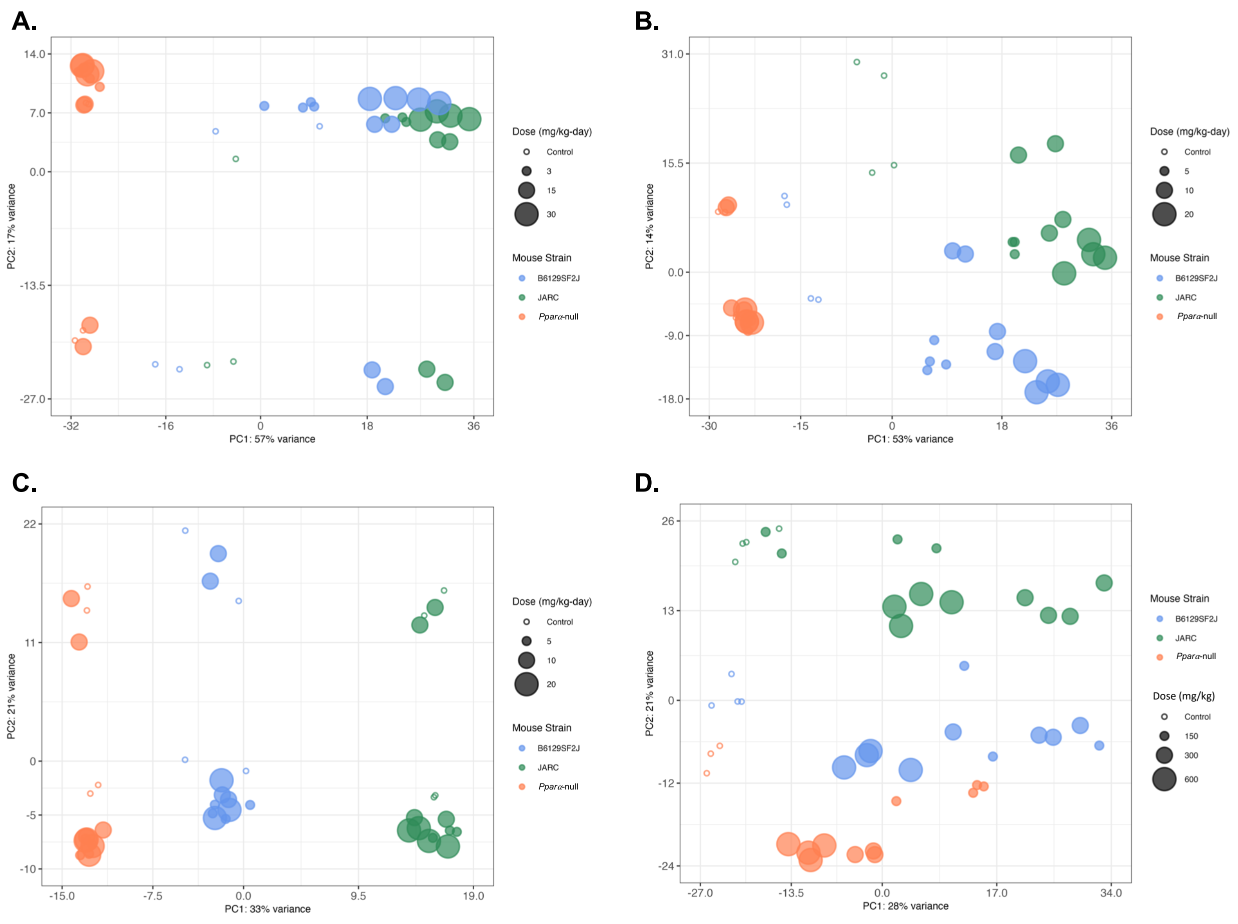

Supplementary Figure S2. Principal component assessment of variance across hepatic transcriptomic profiles for samples within each study arm for HFPO-DA (A), GW7647 (B), rosiglitazone (C), and APAP (D). Each circle represents a sample, with mouse strain and dose level indicated by color and size of each circle.

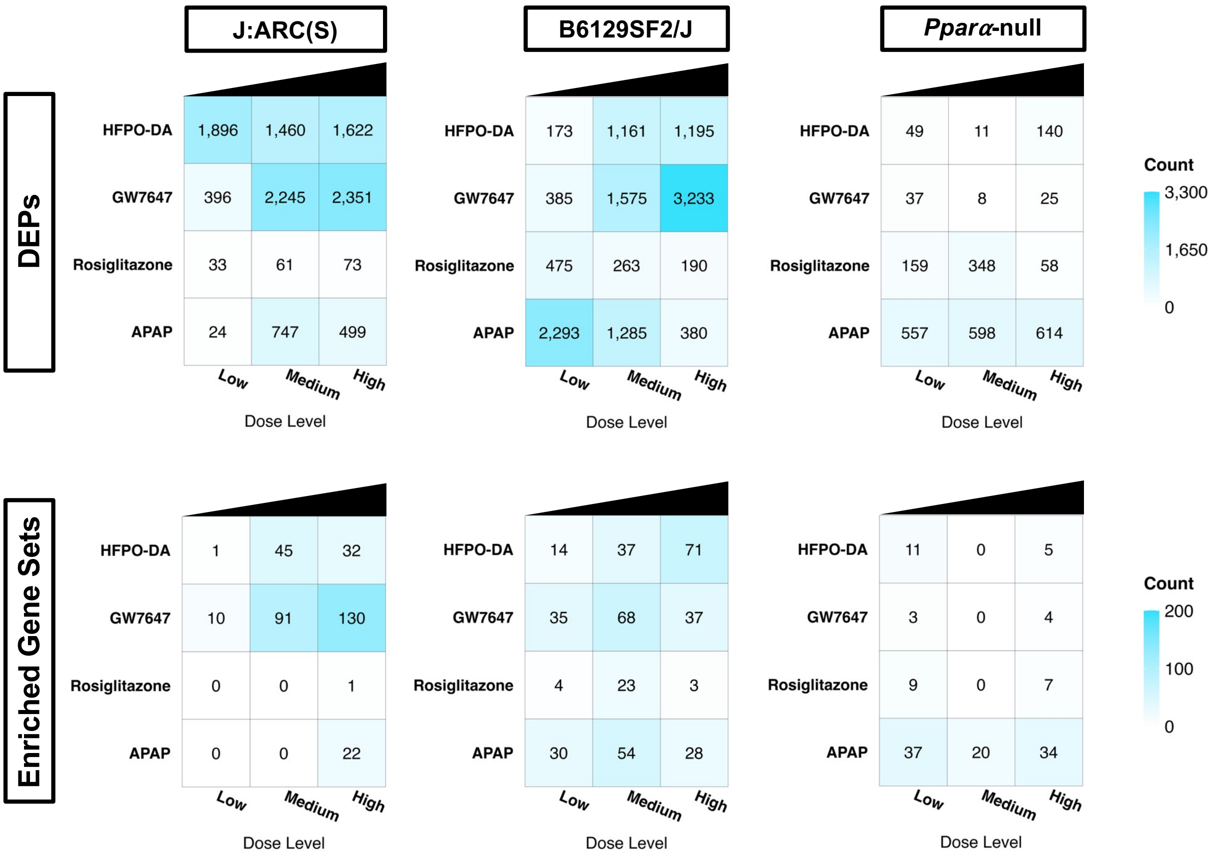

Supplementary Figure S3. Number of significantly downregulated DEPs (relative to controls, FDR<10%, no fold change filter) and enriched gene sets (FDR<5%) in livers from mice exposed to HFPO-DA or a positive control chemical for 5 days or 6 h (APAP only). Each row represents a different chemical, and each column represents a different dose level, with doses increasing from left to right. For APAP-exposed mice, the number of DEPs and enriched gene sets were primarily related to timing of sacrifice (see Supplementary File S1) followed by dose level.

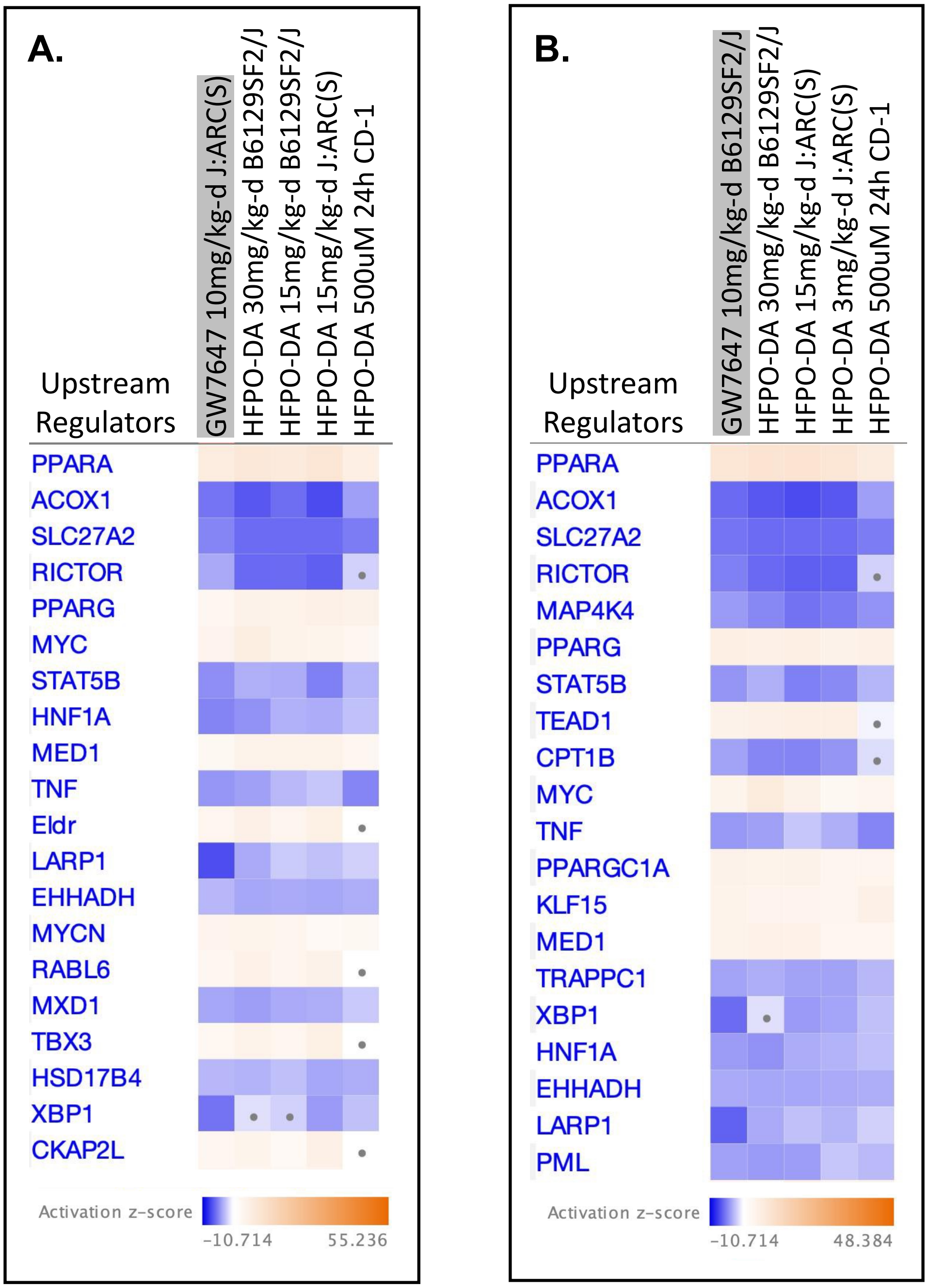

Supplementary Figure S4. Chemical dose groups with the highest overall similarity z-score to J:ARC(S) (A) and B6129SF2/J (B) mice exposed to 10 mg/kg-d GW7647(grey highlight) using IPA match analysis. Each subsequent column after 10 mg/kg-d GW7647 shows the top three analysis matches from the 5-day in vivo study herein in addition to the single top analysis match from previous in vitro transcriptomic studies (Heintz et al. 2024a,b). The overall z-score is calculated using the average scores from IPA enrichment analysis of canonical pathway signatures, predicted upstream regulators, causal networks, and downstream effects. Activation (orange color) and inhibition (blue color) patterns of the top 20 predicted upstream regulators based on z-score are shown in the heatmaps; intensity of each color increases with the absolute z-score. A square containing a dot indicates the z-score did not meet the significance threshold of >|2|.
